# Stomatal conductance, mesophyll conductance, and transpiration efficiency in relation to leaf anatomy in rice and wheat genotypes under drought

**DOI:** 10.1093/jxb/erx314

**Published:** 2017-09-15

**Authors:** Wenjing Ouyang, Paul C Struik, Xinyou Yin, Jianchang Yang

**Affiliations:** 1 Centre for Crop Systems Analysis, Department of Plant Sciences, Wageningen University & Research, AK Wageningen, The Netherlands; 2 College of Agriculture, Yangzhou University, Yangzhou, Jiangsu, China

**Keywords:** Drought, leaf anatomy, mesophyll conductance, rice, stomatal conductance, transpiration efficiency, wheat

## Abstract

Increasing leaf transpiration efficiency (TE) may provide leads for growing rice like dryland cereals such as wheat (*Triticum aestivum*). To explore avenues for improving TE in rice, variations in stomatal conductance (*g*_s_) and mesophyll conductance (*g*_m_) and their anatomical determinants were evaluated in two cultivars from each of lowland, aerobic, and upland groups of *Oryza sativa*, one cultivar of *O. glaberrima*, and two cultivars of *T. aestivum*, under three water regimes. The TE of upland rice, *O. glaberrima*, and wheat was more responsive to the *g*_m_/*g*_s_ ratio than that of lowland and aerobic rice. Overall, the explanatory power of the particular anatomical trait varied among species. Low stomatal density mostly explained the low *g*_s_ in drought-tolerant rice, whereas rice genotypes with smaller stomata generally responded more strongly to drought. Compared with rice, wheat had a higher *g*_m_, which was associated with thicker mesophyll tissue, mesophyll and chloroplasts more exposed to intercellular spaces, and thinner cell walls. Upland rice, *O. glaberrima*, and wheat cultivars minimized the decrease in *g*_m_ under drought by maintaining high ratios of chloroplasts to exposed mesophyll cell walls. Rice TE could be improved by increasing the *g*_m_/*g*_s_ ratio via modifying anatomical traits.

## Teaser

Genotypes of *Oryza sativa*, *O. glaberrima* and *Triticum aestivum* were investigated for leaf gas-exchange parameters in relation to anatomical characteristics. Species-dependent relationships provide leads for growing rice like dryland cereals.

## Introduction

Rice (*Oryza sativa* L.) is a major staple crop that adapts strongly to fully inundated conditions. Compared with other cereal crops, such as wheat (*Triticum aestivum* L.) grown on dry uplands, rice cultivation requires massive amounts of fresh water for irrigation. To minimize the total water requirement for rice cultivation, several water-saving regimes have already been introduced, such as alternate wetting and drying irrigation (Bouman and Tuong, 2001; Zhang *et al.*, 2008) and controlled soil drying during grain filling (Yang *et al.*, 2003; Yang and Zhang, 2006). However, these regimes are largely management measures to reduce water use for rice production. Solving the problem of the intrinsically high water requirement of rice would need breeding or genetic engineering approaches to develop new drought-tolerant rice genotypes with increased water use efficiency (WUE).

Genetic diversity in drought tolerance has long been explored to develop cultivars of *O. sativa* for diverse growing conditions: irrigated lowland, rain-fed lowland, and upland environments. More recently, genotypes suitable for aerobic environments (moderately dry conditions, not inundated lowland or dry upland conditions), i.e. aerobic rice (Bouman *et al.*, 2005; Singh *et al.*, 2008), have been developed. However, aerobic rice cannot replace lowland (‘paddy’) rice in most of the rice-growing areas because of its lower grain yield and is an option only for farmers in rain-fed lowlands with limited or erratic rainfall (Atlin *et al.*, 2006). In addition, African rice (*Oryza glaberrima* Steud.) has evolved as a cultivated species in parallel with *O. sativa*, and is more drought-resistant (Sarla and Swamy, 2005). Natural interspecific hybrids between *O. sativa* and *O. glaberrima* are cultivated in West Africa (Nuijten *et al.*, 2009). The adaptation of rice genotypes to a wider range of edaphic conditions indicates the possibility of growing rice in the same way as other dryland cereal crops such as wheat. Also belonging to the C_3_-crop type, wheat has a relatively higher WUE than rice (Kemanian *et al.*, 2005; Haefele *et al.*, 2009). Compared with rice, wheat shows higher plasticity in root morphological and anatomical adaptation to water-deficit conditions (Kadam *et al.*, 2015). Further comparative analysis of the physiology and anatomy of rice and wheat in response to drought will facilitate the identification of the general traits and mechanisms required for breeding drought-tolerant yet high-yielding rice varieties (Praba *et al.*, 2009).

Photosynthesis is the key process of primary metabolism, and its capacity can influence plant performance and productivity (Lawlor and Tezara, 2009; Pinheiro and Chaves, 2011). Photosynthesis under drought, despite being affected by Rubisco velocity, is often limited by the CO_2_ concentration at carboxylation sites (*C*_c_) inside the chloroplast, which is determined by CO_2_ diffusion components, i.e. stomatal conductance (*g*_s_) and mesophyll conductance (*g*_m_) (Evans *et al.*, 2009). Stomata regulate CO_2_ diffusion into, and water diffusion out of, plant leaves (Chaves *et al.*, 2002). Under water-deficit conditions, plants close stomata to prevent major water loss; this, consequently, reduces photosynthesis via decreased influx of CO_2_ (Pinheiro and Chaves, 2011). In the long-term response to water deficit, *g*_s_ can be influenced by leaf anatomical traits such as stomatal density and size, which can vary to acclimate to the environment (Xu and Zhou, 2008; Franks and Beerling, 2009). As the inevitable consequence of CO_2_ entry through leaf stomata is water loss through transpiration, the stomata-related environmental adaptation may also affect plant instantaneous transpiration efficiency (TE), i.e. the ratio of net photosynthesis rate (*A*_n_) to transpiration rate. In general, higher *g*_s_ results in a lower TE (Flexas *et al.*, 2008; Gu *et al.*, 2012).

Mesophyll conductance (*g*_m_) has been viewed as the diffusion of CO_2_ from sub-stomatal cavities to the sites of carboxylation in the chloroplasts (Flexas *et al.*, 2008). In contrast to *g*_s_, increasing *g*_m_ increases *A*_n_ at no cost of increased transpiration, because the CO_2_ diffusion pathway involving *g*_m_ is not shared with the diffusion pathway of transpired H_2_O. Therefore, increasing *g*_m_ not only increases *A*_n_ but also increases TE (Barbour *et al.*, 2010; Flexas *et al.*, 2013). However, long-term drought stress reduces *g*_m_ (Scartazza *et al.*, 1998; Gu *et al.*, 2012; Han *et al.*, 2016). Along the CO_2_ diffusion pathway inside leaves, the conductance through the liquid phase (*g*_liq_) is the most limiting factor for CO_2_ diffusion in the mesophyll in many species (Gorton *et al.*, 2003; Flexas *et al.*, 2008; Flexas *et al.*, 2012). Growing evidence indicates that (i) genotypic differences in *g*_m_ exist within a given species (Evans and Vellen, 1996; Gu *et al.*, 2012; Jahan *et al.*, 2014), and (ii) leaf mesophyll structure and anatomical properties are important determinants of *g*_m_. Mesophyll thickness (*T*_m_), surface area of chloroplasts exposed to the intercellular airspace (*S*_c_), and mesophyll cell wall thickness (*T*_w_) are suggested to be the most important structural components determining *g*_m_ (Evans *et al.*, 1994; Evans *et al.*, 2009; Scafaro *et al.*, 2011; Tosens *et al.*, 2012*b*; Tomás *et al.*, 2013), as these parameters determine pathways for CO_2_ permeation into chloroplasts (Terashima *et al.*, 2011). Therefore, investigating relationships between leaf anatomy and photosynthetic features could improve understanding of leaf structural features required to enhance drought tolerance in rice.

In this study, the photosynthetic diffusion components (*g*_s_ and *g*_m_) and possibly related leaf anatomical properties were examined in three species, *O. sativa*, *O. glaberrima* and *T. aestivum*. *g*_s_ was measured from gas exchange, and combined gas exchange and chlorophyll fluorescence measurements were used to estimate *g*_m_. Leaf anatomical properties were examined through light and transmission electron microscopy. The objectives of this study were: (i) to evaluate the variation in photosynthetic capacity among the contrasting types of rice species as well as between rice and wheat in response to a long-term drought, and (ii) to assess the leaf anatomical determinants of *g*_s_ and *g*_m_ and how those determinants underpin genotypic differences in the response of *g*_s_, *g*_m_, and TE under drought.

## Materials and methods

### Plant materials and treatments

Six cultivars from *O. sativa*, one cultivar from *O. glaberrima*, and two cultivars from *T. aestivum* were used for the study of photosynthetic and leaf anatomical properties. The *O. sativa* cultivars represented rice types bred for lowland, aerobic or upland cultivation systems, respectively, whereas wheat cultivars were selected based on their drought tolerance (Table 1). The tolerance and susceptibility classifications were based on previous reports (Thanh *et al.*, 1999; George *et al.*, 2002; Sarla and Swamy, 2005; Atlin *et al.*, 2006; Liu *et al.*, 2006; Praba *et al.*, 2009). Seeds of rice and wheat cultivars were obtained from the International Rice Research Institute (IRRI) and from the International Maize and Wheat Improvement Center (CIMMYT), respectively.

**Table 1.** Description of rice and wheat cultivars used in the study

Species	Cultivar	Abbrev.	Cultivar type	Drought tolerance
*Oryza sativa*	IR 64-21	IR64	High-yielding lowland cultivar	Susceptible
	IR 77298-14-1-2::IRGC 117374-1	II	Lowland cultivar	Susceptible to moderately tolerant
	NSIC RC 9	Apo	High-yielding aerobic cultivar	Moderately tolerant
	NSIC RC 192	148	High-yielding aerobic cultivar	Moderately tolerant
	UPL Ri7	UPL7	Improved upland cultivar	Drought tolerant
	Salumpikit	Sal	Traditional upland cultivar	Drought tolerant
*Oryza glaberrima*	CG14	CG14	Cultivated African rice	Drought tolerant
*Triticum aestivum*	SeriM82	S82	High-yielding irrigated cultivar	Moderately susceptible
	Weebill4	We4	Dryland-adapted cultivar	Drought tolerant

Pot experiments were conducted at Yangzhou University, Jiangsu Province, China (32°30′ N, 119°25′ E). Rice seeds were sown in saturated soil after pre-germination, and wheat seeds were sown directly in moist soil. The pots were placed in an open field sheltered from rain by a mobile transparent polyethylene shelter. Each pot (30 cm in height, 25 cm in diameter, 14.72 liters in volume) contained 20 kg of sandy loam soil from the Yangzhou University rice/wheat rotated experimental field. One day before sowing, 1.1 g CO(NH_2_)_2_ (urea) and 0.5 g KH_2_PO_4_ were pre-mixed through the soil per pot. Extra nitrogen (2.5 g urea for rice and 1.5 g urea for wheat per pot) were split-applied during different plant growth stages.

We imposed three levels of soil moisture, i.e. control (CT), mild drought stress (MD), and more severe drought stress (SD). Across all species and treatments, three replications were maintained and pots were placed in a randomized design. Soil water potential was monitored by inserting a tension meter (Institute of Soil Sciences, Chinese Academy of Sciences, Nanjing, China) at 15 cm soil depth throughout the experiments. Because rice and wheat have different growth duration and are naturally adapted to different moisture environments during domestication histories (Praba *et al.*, 2009), different timings and intensity of stress impositions were applied (Table 2). When tension-meter readings reached the lower limit in each stress level, tap water was added until the upper limit of the target stress was reached. Once stress was imposed, the target stress levels were maintained to ensure that leaves for measurements (see below) were initiated and developed under stress, until all the measurements were completed.

**Table 2.** Description of cultivation and water regimes for rice and wheat cultivars CT, control; MD, mild drought; SD, more severe drought.

Species	Sowing time	Density (seedlings per pot)	Soil pH	Initial soil moisture	Stress imposed time (leaves)^*a*^	Water stress level			Final leaf number^*b*^
						CT	MD	SD	
*O. sativa,* *O. glaberrima*	June 2013	3	5.5–6.0	Saturated	5	Inundated	0 to −5 kPa	−20 to −40 kPa	14–15
*T. aestivum*	November 2013	8	Not adjusted	0 to −5 kPa	4	0 to −5 kPa	−20 to −40 kPa	−50 to −70 kPa	10–11

^*a*^ Leaf number on the main stem.

^*b*^ The final leaf number for the flag leaf on the main stem; this number was 18 for cv. Sal of *O. sativa*.

### Gas exchange and chlorophyll fluorescence measurements

An open gas exchange system integrated with a fluorescence chamber head (Li-Cor 6400XT; Li-Cor Inc., Lincoln, NE, USA) was used to simultaneously measure gas exchange (GE) and chlorophyll fluorescence (CF) parameters. During flowering, the penultimate leaf on the main shoot from each replication per treatment was used for measurements. To avoid the effect of fluctuation in outdoor environment on GE measurement, all measurements were taken in a climate chamber with air temperature at 28 °C (23 °C for wheat), 65% relative humidity and a photosynthetic photon flux density at the leaf surface of 1200 μmol m^–2^ s^–1^ (artificial light source). All measurements were made at a leaf temperature of 25 °C and the leaf-to-air vapor pressure difference (VPD) was kept between 1.0 and 1.6 kPa. Light and CO_2_ response curves were measured under both ambient (21%) and low (2%) O_2_ conditions. The low O_2_ condition was created by using a gas mixture of 2% O_2_ and 98% N_2_, and the infrared gas analyser calibration was adjusted for O_2_ composition of the gas mixture according to the manufacturer’s instructions.

For the CO_2_ response curve, under both O_2_ conditions, the leaf was consecutively exposed to an incident irradiance (*I*_inc_) of 1000 µmol m^−2^ s^−1^ with different levels of CO_2_: 50, 90, 150, 250, 400, 700, 1000, and 1500 µmol mol^−1^. For the light response curve, under the 21% O_2_ condition, the CO_2_ was kept constant at 400 µmol mol^−1^ and *I*_inc_ was increased in the order of 30, 50, 80, 120, 200, 500, 1000, and 1600 µmol m^−2^ s^−1^. Under 2% O_2_, in order to ensure non-photorespiratory conditions, the CO_2_ was kept constant at 1000 µmol mol^−1^ with *I*_inc_ levels of 30, 50, 80, 120, and 200 µmol m^−2^ s^−1^. The measurement flow rate was 400 μmol s^−1^. CO_2_ exchange rates were corrected for CO_2_ leakage into and out of the leaf cuvette, based on measurements using the same flow rate on boiled leaves across a range of CO_2_ levels, and intercellular CO_2_ levels (*C*_i_) were then recalculated (Flexas *et al.*, 2007).

The steady-state fluorescence (*F*_s_) was measured at each light or CO_2_ step. Then a multiphase flash method (Loriaux *et al.*, 2013) was applied to determine *F*′_m_ (the maximum fluorescence during the saturating light pulse). The apparent photosystem II electron (e^−^) transport efficiency for each irradiance or CO_2_ step was calculated as Ф_2_=(*F*′_m_–*F*_s_)/*F*′_m_ (Genty *et al.*, 1989).

### Estimation of diffusion components

Total conductance to CO_2_ (*g*_tot_) was calculated based on stomatal and mesophyll conductance according to 1/*g*_tot_=1/*g*_s_+1/*g*_m_. For the analysis, stomatal conductance for CO_2_ (*g*_s_) was obtained as stomatal conductance for water vapour divided by 1.6, from the data points measured under ambient condition (400 µmol mol^−1^ CO_2_, 1000–1500 µmol m^−2^ s^−1^ irradiance, and 25 °C) of the light response curve under 21% O_2_.

In order to compare *g*_m_ across species, genotypes, and treatments, the value of *g*_m_ assumed as constant was estimated using the NRH-A variant method (Yin and Struik, 2009):

$$A_{n}=0.5\{J/4-R_{d}+g_{m}(C_{i}+2\Gamma^{{}_{*}})-\sqrt{\begin{array}{l} {[}^{J/4-R_{d}+g_{m}(C_{i}+2\Gamma^{{}_{*}})]2}-\\ 4g_{m}[(C_{i}-\Gamma^{{}_{*}})J/4-R_{d}(C_{i}+2\Gamma^{{}_{*}})] \end{array}}\}$$ (1)

where Γ* is the CO_2_ compensation point in the absence of day respiration (*R*_d_) and *J* is the linear electron transport rate used for CO_2_ fixation and photorespiration. Equation (1) provides a model to estimate *g*_m_ by a curve fitting that minimizes the difference between measured and estimated *A*_n_ (Yin and Struik, 2009). In the model, Γ* is calculated from the Rubisco specificity factor (*S*_c/o_) as Γ*=0.5*O*/*S*_c/o_, where *O* is O_2_ partial pressure (mbar). *S*_c/o_ at a given temperature is expected to be constant for a specific species; however, the value at 25°C did not differ much between rice and wheat (Makino *et al.*, 1988; Yin *et al.*, 2009; Gu *et al.*, 2012), so a single value for *S*_c/o_ at 25 °C (3.13 mbar µbar^−1^) was adopted from Makino *et al.* (1988) and Yin *et al.* (2009). Data obtained from low *I*_inc_ levels of the light response curve and high *C*_a_ levels of the CO_2_ response curve measured at 21% O_2_ were used for the curve fitting. Model inputs (*J* and *R*_d_) were estimated as described by Yin *et al.* (2009). In brief, using data of light-limited range under non-photorespiratory conditions (i.e. the light response curve under 2% O_2_ with 1000 µmol mol^−1^ CO_2_ plus points from >500 µmol mol^−1^*C*_a_ levels of CO_2_ response curve under 2% O_2_), a linear regression can be performed for the observed *A*_n_ against *I*_inc_Ф_2_/4. The day respiration (*R*_d_) is the intercept of the linear regression, and the slope of the regression yields the estimated lumped parameter *s* (Yin *et al.*, 2009). Then *J* at each *C*_i_ can be calculated using *J=sI*_inc_Ф_2_.

### Light and transmission electron microscopy

Stomatal density and size were determined using the silicon rubber impression technique (Smith *et al.*, 1989). Stomatal features from this method did not differ from those based on glancing sections cut from fresh leaf sample. The method and impression material are described by Giday *et al.* (2013). Imprints were taken from both adaxial and abaxial surfaces of the area where GE and CF were measured, and were later on smeared with nail polish in the mid-area between the central vein and the leaf edge, for approximately 20 min. The thin film was carefully peeled off the imprint with no stretching and mounted on a glass slide with a drop of water to improve contrast. Stomatal density (*D*) was determined by counting the maximum numbers of stomata from ten randomly selected non-overlapping rectangular fields of view per leaf under the light microscope (Axio Imager D2, Carl Zeiss, Germany). Stomatal size (*S*) was calculated by multiplying guard cell length (*L*) by width (*W*). All parameters were determined as the average of both sides of the leaf. Specific stomatal conductance (*sg*_s_) was determined by dividing *g*_s_ by *D* (Ohsumi *et al.*, 2007). An integrative parameter stomatal area index (SAI) was calculated by multiplying density by size and expressed in mm^2^ stomata mm^−2^ leaf. Another integrative parameter, so-called maximum stomatal diffusive conductance (*g*_smax_), was also calculated, according to Franks and Beerling (2009):

$$g_{\text{s}\max}=\frac{d\cdot D\cdot a_{\max}}{1.6v\left(l+\frac{\pi }{2}\sqrt{a_{\max}/\pi }\right)}$$ (2)

where *d* is the diffusivity of water vapour in air, *v* is the molar volume of air, *a*_max_ is the maximum area of the open stomatal pore, and *l* is the stomatal pore depth for fully open stomata. The values for standard gas constants *d* and *v* at 25 °C are 2.82 × 10^–5^ m^2^ s^−1^ and 24.5 m^3^ mol^−1^, respectively. *a*_max_ was calculated as π(*p*/2)^2^, where *p* is the stomatal pore length. *p* was calculated as *L*/2 according to Franks and Beerling (2009). *l* was taken as equal to *W*/2, assuming guard cells inflate to circular cross section.

For the anatomical measurement of mesophyll cells, leaflet samples were collected from the leaves where GE and CF were measured. Half of the leaves were fixed and further used for anatomical study. Tissue fixation and preparation for light and transmission electron microscopy (TEM) studies followed the method of Scafaro *et al.* (2011) with small modification. Small leaf samples (4 × 1.5 mm^2^) from each cultivar×water treatment combination were cut parallel to the main vein avoiding large veins. The samples were infiltrated in a solution of 3% glutaraldehyde, 2% paraformaldehyde, and 0.1 M phosphate buffer (pH 7.2) for at least 48 h. Samples were further post-fixed in 1% osmium tetroxide for 2 h, followed by dehydration in an ethanol series (30, 50, 70, 80, 90, 95, and 100%). Ethanol was replaced by 1,2-epoxypropane, and the samples were further embedded with Spurr’s resin (London Resin Company, London, UK) and cured in an oven at 70 °C for 12 h. Transverse sections for light and TEM studies were cut using an ultra-microtome (Ultra-cut R, Leica, Germany). Leaf cross sections 1 µm thick were cut for the light microscopy study, and sections 70 nm thick were cut for the TEM study. Light sections were stained with safranin (1%) for 20 s and followed with methyl purple (1%) for 20 s, then viewed and photographed under a light microscope (Axio Imager D2, Carl Zeiss, Germany). Electron sections were doubly stained with uranyl acetate for 30 min and lead citrate for 15 min, and then photographed under a transmission electron microscope (Tecnai 12, Philips, The Netherlands).

Analyses of images were performed using ImageJ software (National Institutes of Health, Bethesda, MD, USA) (Abràmoff *et al.*, 2004). All mesophyll anatomical characteristics were determined in multiple sections, with 12–30 complete mesophyll cells per replication per treatment depending on the image quality. The analysis protocol followed the method of Evans *et al.* (1994). Mesophyll thickness (*T*_m_) was measured as the length between the two epidermises of the leaf from light microscopy images. The surface area of mesophyll cells exposed to the intercellular airspace per leaf area (*S*_m_) was calculated from light microscopy images as:

$$S_{\text{m}}=\frac{L_{\text{m}}}{w}F$$ (3)

where *w* is the width of the section measured, *L*_m_ is the length of mesophyll exposed to the intercellular airspace, and *F* is the curvature correction factor (Thain, 1983; Evans *et al.*, 1994). Although the mesophyll size differed between rice and wheat, mesophyll cells in both species are presented as lobed spheroids with a different orientation of the long axes to the veins (Chonan, 1970, 1972; Sage and Sage, 2009), and thus a difference of curvature correction factors between species was not taken into account in this study, and we adopted the value 1.55 from a previous rice study (Scafaro *et al.*, 2011). Uncertainties were also analysed given that Barbour *et al.* (2016) recently assumed a value of 1.25 for wheat.

The chloroplast surface area exposed to the intercellular airspace per leaf area (*S*_c_) was calculated as:

$$S_{\text{c}}=\frac{L{'}_{\text{c}}}{L{'}_{\text{m}}}S_{\text{m}}$$ (4)

where *L*′_c_ is the length of chloroplast exposed to the intercellular airspace and *L*′_m_ is the corresponding length of mesophyll exposed to the intercellular airspace. *L*′_c_ and *L*′_m_ were determined from TEM images based on average values measured for 12–30 mesophyll cells (parameters obtained from TEM are marked by a prime (′) to distinguish them from *L*_m_ measured from light microscopy images). The ratio of the exposed surface area of chloroplast to the exposed surface area of mesophyll cell walls (*S*_c_/*S*_m_) was set equal to *L*′_c_/*L*′_m_ (Tosens *et al.*, 2012*a*,b).

In addition, thickness of the mesophyll cell wall (*T*_w_) was also determined from TEM images. Of the thickness values measured for different sections of each mesophyll cell, only the lower half of the values were averaged as the *T*_w_ of that cell to minimize the artefact of inaccurate orientation of ultra-microtome sectioning. The average value of *T*_w_ from all mesophyll cells in each cultivar and treatment was used for further analysis.

### N content measurements

The other half of the collected leaflets after GE and CF measurements were used to measure the leaf area and then oven dried at 70 °C to constant weight. Leaflets were weighed to determine leaf mass per unit area (LMA), and then ground to fine powder. Samples were analysed for N content by using an elementary analyser (Vario Macro cube, Elementar, Germany). Leaf nitrogen per unit area (*N*_a_) was calculated from these data as a measure of leaf physiological status.

### Statistical analyses

A one-way analysis of variance (ANOVA) was used to reveal the differences between cultivars in the studied characteristics. A two-way ANOVA was used to study the effect of genotypes, drought treatment, and their interaction on photosynthetic and anatomical parameters. Linear regression analyses were also conducted. These analysis were performed using the R programming language (http://www.R-project.org/). Non-linear fitting for Eq. (1) was carried out using the GAUSS method in PROC NLIN of SAS (SAS Institute Inc., Cary, NC, USA).

## Results

### Variation in photosynthesis

**Fig. 1.**
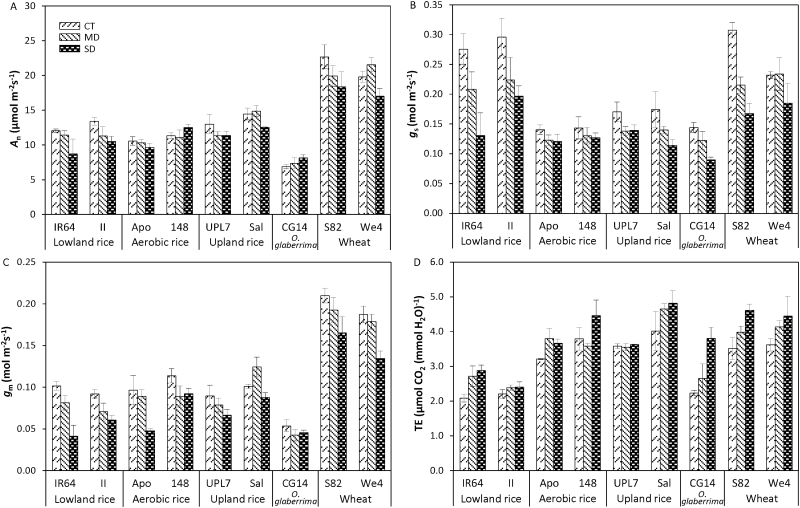
Photosynthetic parameters obtained and estimated from GE and CF data under three treatments: control (CT), mild drought (MD), and more severe drought (SD). Values of (A) net photosynthetic rate (*A*_n_), (B) stomatal conductance (*g*_s_), and (D) transpiration efficiency (TE) were obtained under the condition of 400 µmol mol^−1^ CO_2_, 1000–1500 µmol m^−2^ s^−1^ irradiance, and 25 °C. (C) Mesophyll conductance (*g*_m_) was calculated based on the NRH-A method (Yin and Struik, 2009). Bars represent standard errors of the mean for *A*_n_, *g*_s_, and TE, and standard error of the estimate for *g*_m_.

The net photosynthesis rate (*A*_n_) varied almost threefold among species, with *T. aestivum* having a significantly higher *A*_n_ than *O. sativa* and *O. glaberrima* (cv. CG14) (Fig. 1A). Under the CT condition, *A*_n_ varied between 6.9 µmol m^−2^ s^−1^ for CG14 and 22.7 µmol m^−2^ s^−1^ for S82. Overall, drought decreased *A*_n_ strongly with no significant interaction between cultivars and water treatments (Supplementary Table S1 at *JXB* online). However, for CG14, drought increased *A*_n_ (Fig. 1A), and this was probably associated with an increase in *N*_a_ by drought in this cultivar (Supplementary Fig. S1A). Across all cultivars and treatments, *A*_n_ was positively correlated with *N*_a_ among wheat cultivars, but not among cultivars of *O. sativa* (Supplementary Fig. S1B).

### Variation in *g*_s_ and *g*_m_

Significant variation in stomatal conductance (*g*_s_) was observed both among species and within *O. sativa* (Supplementary Table S1). Notably, lowland rice and wheat cultivars had a much higher *g*_s_ than other cultivars (Fig. 1B). *g*_s_ decreased under drought in all cultivars, with mild interaction between cultivars and water treatments (0.05<*P*<0.1). The decrease in *g*_s_ was stronger in IR64, Sal, CG14, and S82 than in other cultivars (Fig. 1B). Mesophyll conductance (*g*_m_) varied significantly among species, with wheat having a higher *g*_m_ than *O. sativa*, and CG14 having the lowest value (Fig. 1C). In wheat, the drought effect on *g*_m_ was only significant in We4 under SD condition (28% reduction). The differences in *g*_m_ among *O. sativa* cultivars increased with an increase in stress level (*P*>0.05 under CT, *P*<0.05 under MD, and *P*<0.01 under SD). Only lowland rice cultivars had significant decreases in *g*_m_ under the MD condition. Compared with *g*_m_ in CT, a significant decrease in *g*_m_ by SD was observed in lowland rice and aerobic rice cv. Apo, but not in other cultivars. The lowest decrease in *g*_m_ was observed in Sal and CG14 (*ca* 14%), and the highest decrease was recorded in IR64 (59%).

### Influences of *g*_s_ and *g*_m_ on *A*_n_ and transpiration efficiency

**Fig. 2.**
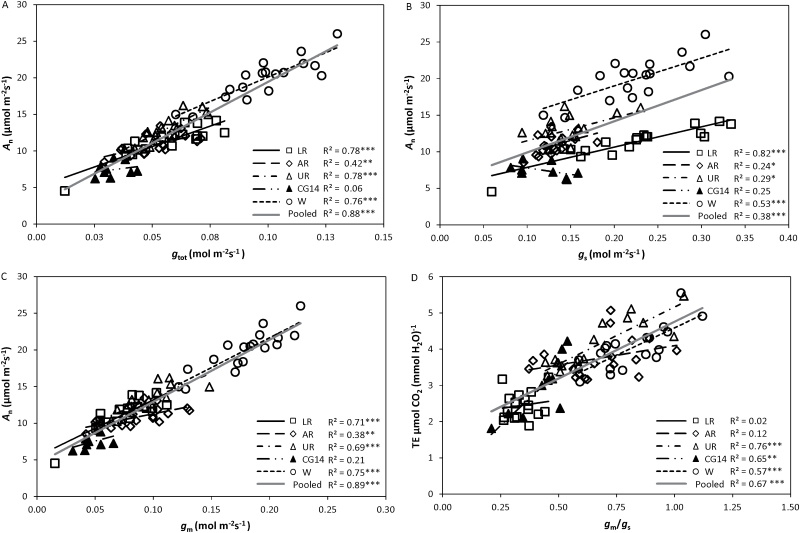
The relationships (A) between photosynthetic rate (*A*_n_) and total leaf conductance (*g*_tot_), (B) between *A*_n_ and stomatal conductance (*g*_s_), (C) between *A*_n_ and mesophyll conductance (*g*_m_), and (D) between transpiration efficiency (TE) and the ratio of mesophyll conductance to stomatal conductance (*g*_m_/*g*_s_). *A*_n_, *g*_s_, and TE were obtained under 400 µmol mol^−1^ CO_2_, 1000–1500 µmol m^−2^ s^−1^ light intensity, and 25 °C. *g*_m_ was calculated based on the NRH-A method (Yin and Struik, 2009). AR, aerobic rice; CG14, *O. glaberrima*; LR, lowland rice; UR, upland rice; W, wheat. Linear regressions were fitted for overall data and for each genotype group. The significance of each correlation is shown by asterisks: *P<0.05, **P<0.01, ***P<0.001.

Significant positive correlations between *A*_n_ and total conductance (*g*_tot_) were observed among species and within each group (Fig. 2A), indicating the presence of major diffusion limitations to CO_2_ assimilation. Different individual effects of *g*_s_ and *g*_m_ on *A*_n_ were observed (Fig. 2B, C). The correlation between *A*_n_ and *g*_s_ was highly significant in lowland rice, in the wheat group, and for all data combined (*P*<0.001), and weaker in aerobic and upland rice (*P*<0.05) and in *O. glaberrima* (*P*>0.05) (Fig. 2B). On the other hand, the correlation between *A*_n_ and *g*_m_ was highly significant for all data combined as well as in most of the subgroups (Fig. 2C). Multiple regression analysis indicated that *g*_s_ contributed more to the variation in *A*_n_ in lowland rice cultivars, whereas *g*_m_ contributed more to the variation in *A*_n_ in aerobic rice, upland rice, and wheat cultivars (Supplementary Table S2).

Aerobic rice, upland rice, and wheat cultivars had higher TE than lowland rice cultivars (Fig. 1D). Under the CT condition, TE varied between 2.09 µmol CO_2_ (mmol H_2_O)^−1^ for IR64 and 4.02 µmol CO_2_ (mmol H_2_O)^−1^ for Sal. TE increased under drought, and the highest increase in TE was recorded for CG14 under the SD condition, with a 70% increase over the CT condition. A weak negative correlation between TE and *g*_s_ was observed within each genotype group (Supplementary Fig. S2A), but the correlation between TE and *g*_m_ was not significant in most groups (Supplementary Fig. S2B). However, TE was well explained by combined *g*_s_ and *g*_m_ (Supplementary Table S3) and was actually highly correlated to the *g*_m_/*g*_s_ ratio (Fig. 2D), especially in upland rice, CG14, and wheat cultivars, although this relationship was less clear in lowland and aerobic rice cultivars. Both the *g*_m_/*g*_s_ and the TE of aerobic rice, upland rice, and wheat cultivars were higher than those of lowland rice cultivars.

### Anatomical determinants of *g*_s_

**Fig. 3.**
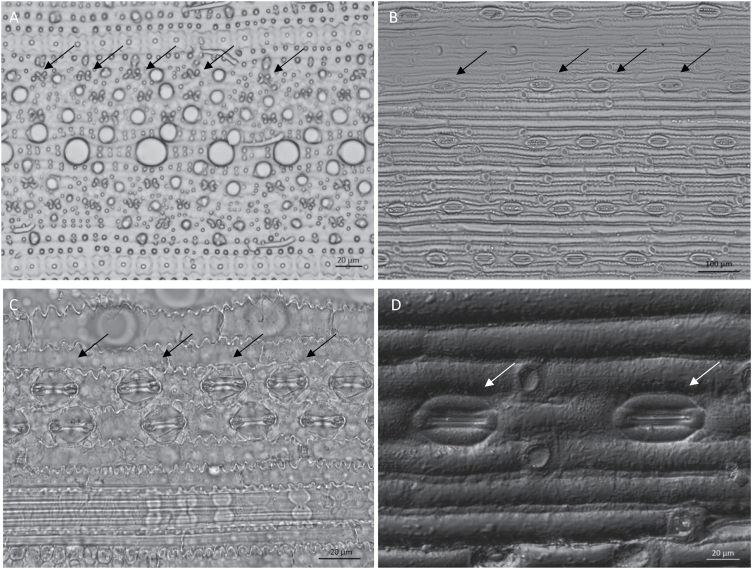
Light micrographs illustrating leaf surfaces of rice (IR64; A, C) and wheat (S82; B, D) used to calculate stomatal density (A, B) and size (C, D). Arrows indicate stomata.

Wheat cultivars had significantly lower density and larger stomata than rice cultivars (Fig. 3 and Table 3). Stomatal density (*D*) varied significantly among rice cultivars, with lowland rice having higher *D* than aerobic and upland rice, and CG14 having the lowest value. In rice, *D* on the adaxial side (*D*_adaxial_) was lower than that on the abaxial side (*D*_abaxial_), and *D*_adaxial_ varied significantly among cultivars, whereas less variation was observed for *D*_abaxial_ (Table 3). Interestingly, the drought effect was significant only for *D*_adaxial_ but not for *D*_abaxial_ in both rice and wheat (Supplementary Table S1). Drought increased *D*_adaxial_ in most cultivars (Supplementary Fig. S3A).

**Table 3.** *Leaf anatomical properties of* O. sativa, O. glaberrima*, and* T. aestivum *under the control (CT) condition* Mesophyll thicknesses (*T*_m_), mesophyll surface area exposed to intercellular airspace (*S*_m_), and stomatal size (*S*) and density (*D*) were determined from light microscope images. The ratio of the exposed chloroplast surface area to the exposed surface area of mesophyll cell walls (*S*_c_/*S*_m_) and mesophyll cell wall thickness (*T*_w_) were determined from transmission electron microscope images. Chloroplast surface area exposed to intercellular airspace (*S*_c_) was obtained by multiplying *S*_m_ by *L*′_c_/*L*′_m_. Data are means±standard error of three replicates. The significance of differences between cultivars was based on a one-way analysis of variance. The significance level of overall data/within *O. sativa* is shown by asterisks: *P<0.05, **P<0.01, ***P<0.001.

Species	Genotype group	Cultivar	Mesophyll					Stomata			
			*T*_m_ (µm)	*S*_m_ (m m^−2^)	*S*_c_/*S*_m_	*S*_c_ (m m^−2^)	*T*_w_ (nm)	*S* (µm^2^)		*D* (No. mm^−2^)	
								Adaxial	Abaxial	Adaxial	Abaxial
*O. sativa*	Lowland rice	IR64	70.9 ± 4.2	11.2 ± 0.2	0.77 ± 0.01	8.7 ± 0.3	126 ± 1	172.1 ± 2.1	186.7 ± 2.5	621.0 ± 7.6	732.7 ± 15.1
		II	91.6 ± 0.6	10.8 ± 0.2	0.71 ± 0.02	7.6 ± 0.1	151 ± 2	194.7 ± 10.3	203.5 ± 4.0	547.1 ± 5.8	680.9 ± 15.1
	Aerobic rice	Apo	73.6 ± 0.9	10.6 ± 0.3	0.68 ± 0.02	7.2 ± 0.4	136 ± 3	217.8 ± 11.0	215.6 ± 11.3	460.0 ± 26.1	678.3 ± 10.2
		148	70.1 ± 1.5	9.7 ± 0.3	0.72 ± 0.02	7.0 ± 0.2	140 ± 4	211.3 ± 7.0	213.0 ± 6.1	509.1 ± 17.9	639.4 ± 22.4
	Upland rice	UPL7	82.4 ± 1.7	10.9 ± 0.2	0.71 ± 0.02	7.7 ± 0.2	174 ± 2	188.7 ± 6.3	186.8 ± 5.4	475.6 ± 36.4	640.8 ± 41.6
		Sal	79.7 ± 0.7	11.5 ± 0.1	0.87 ± 0.02	10.1 ± 0.3	182 ± 1	185.3 ± 5.6	168.7 ± 4.5	467.4 ± 15.2	685.4 ± 21.7
*O. glaberrima*	African rice	CG14	59.8 ± 1.8	11.9 ± 0.4	0.62 ± 0.03	7.4 ± 0.3	161 ± 4	183.5 ± 8.1	174.9 ± 11.6	361.5 ± 34.3	498.0 ± 52.7
*T. aestivum*	Wheat	S82	164.7 ± 1.2	14.3 ± 1.1	0.81 ± 0.03	11.6 ± 1.0	110 ± 2	1592.4 ± 42.9	1755.5 ± 46.0	67.8 ± 0.6	58.7 ± 1.1
		We4	147.2 ± 2.2	14.9 ± 0.9	0.87 ± 0.01	13.0 ± 0.9	116 ± 2	1600.2 ± 32.9	1692.5 ± 72.7	61.7 ± 2.0	45.1 ± 1.8
*P*-value			***/***	***/*	***/***	***/***	***/***	***/*	***/**	***/**	***/ns

Aerobic rice cultivars had clearly larger stomatal size (*S*) than other rice cultivars (Table 3). This high *S* resulted more from wider stomatal width (*W*) than from stomatal length (*L*) (data not shown). Drought decreased *S*, and the effect was similar on both leaf surfaces in all cultivars (Supplementary Fig. S3C, D). Cultivars CG14 and S82 had the strongest decrease in *S* in rice and wheat, respectively.

**Fig. 4.**
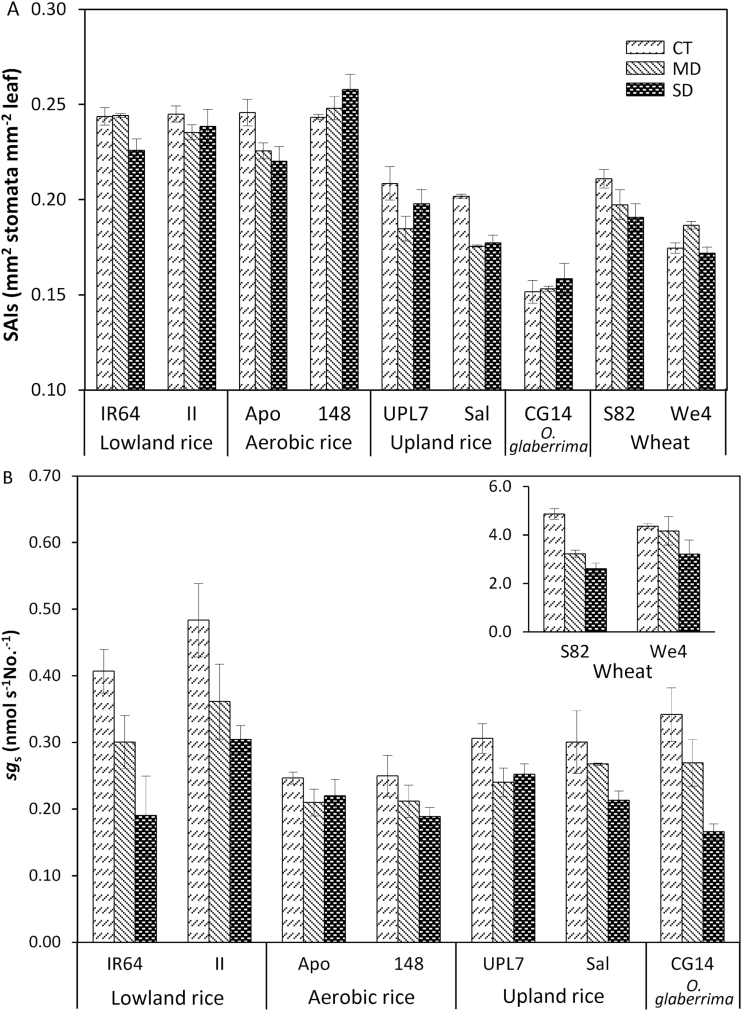
Response of (A) the summed stomatal area index on both sides of the leaf surface (SAIs) and (B) specific stomatal conductance (*sg*_s_) of rice and wheat cultivars to water treatments: control (CT), mild drought (MD), and more severe drought (SD).

The sum of the stomatal area index on both sides of the leaf (SAIs) differed among the three species, with CG14 having the lowest SAIs and lowland, aerobic rice having higher SAIs than the other cultivars. The variation in SAIs within rice species was more associated with *D* (*R*^2^=0.58) than with *S* (*R*^2^=0.33). SAIs decreased under drought in most cultivars, except in 148, CG14 and We4 (Fig. 4A). Specific stomatal conductance (*sg*_s_) was much higher in wheat than in rice cultivars, and higher in lowland rice than in other rice cultivars under the CT condition (Fig. 4B). Drought decreased *sg*_s_ in all cultivars, with IR64, CG14, and S82 showing the highest decrease among rice and wheat cultivars, respectively.

**Fig. 5.**
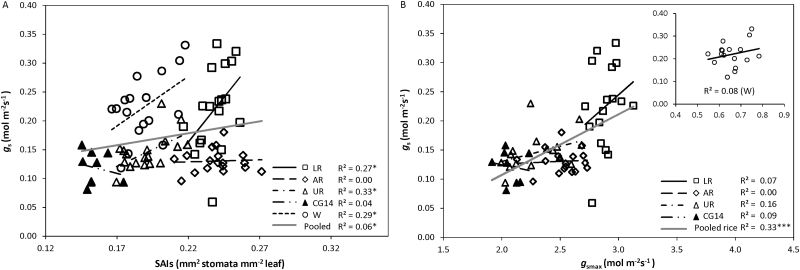
The relationships (A) between stomatal conductance (*g*_s_) and the summed stomatal area index (SAIs), and (B) between *g*_s_ and the maximum stomatal diffusive conductance (*g*_smax_). Values of *g*_s_ were obtained and calculated under 400 µmol mol^−1^ CO_2_, 1000–1500 µmol m^−2^ s^−1^ light intensity, and 25 °C. AR, aerobic rice; CG14, *O. glaberrima*; LR, lowland rice; UR, upland rice; W, wheat. Linear regressions were fitted for overall data and for each genotype group. The significance of each correlation is shown by asterisks: *P<0.05, **P<0.01, ***P<0.001.

The variation in stomatal properties can partially explain the variation in *g*_s_ among species and within species. *g*_s_ was partially correlated with *S* or *D*, depending on genotype groups (Supplementary Fig. S4). A significant positive correlation was found between *g*_s_ and SAIs for lowland and upland *O. sativa* as well as for wheat genotypes (Fig. 5A). The theoretical maximum stomatal conductance (*g*_smax_) was higher than the measured *g*_s_ in all cultivars and treatments, and was higher in rice than in wheat (Fig. 5B). The correlation between *g*_s_ and *g*_smax_ was significant only for *O. sativa*, and not in wheat and each genotype group (Fig. 5B).

### Anatomical determinants of *g*_m_

**Fig. 6.**
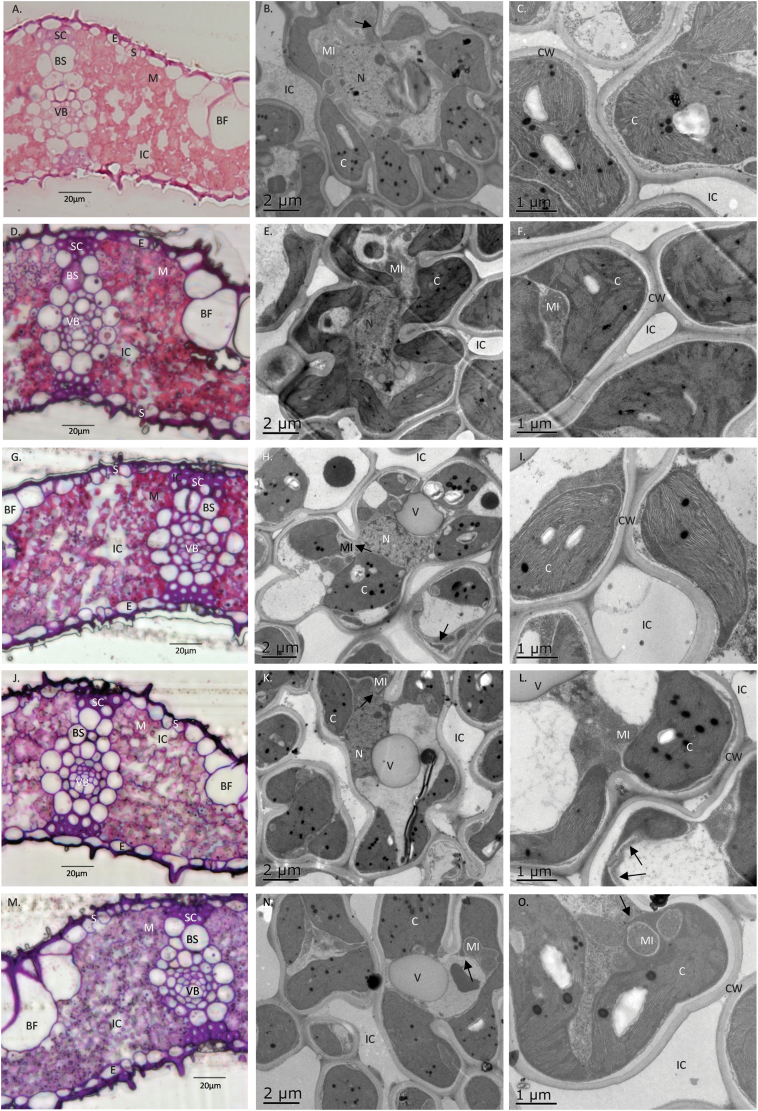

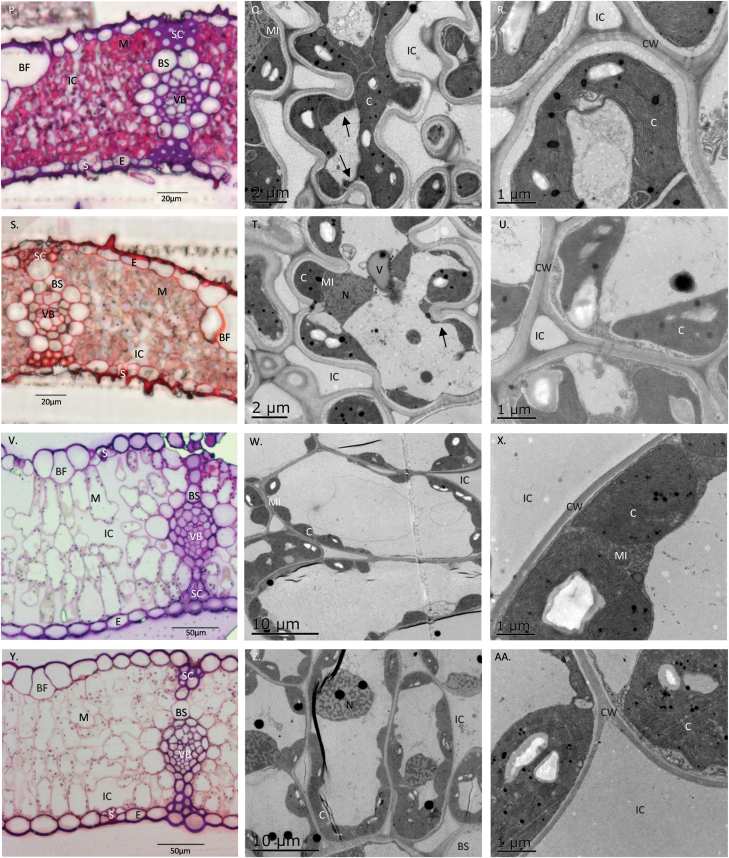
Light (left panels) and electron (middle and right panels) micrographs of leaf structure and anatomy for *O. sativa* cultivars (A–C, IR64; D–F, II; G–I, Apo; J–L, 148; M–O, UPL7; P–R, Sal), *O. glaberrima* (S–U, CG14), and *T. aestivum* cultivars (V–X, S82; Y–AA, We4). The left panels show the overall leaf transverse sections; the middle panels show mesophyll cell shape, chloroplast distribution, and lobe development; and the right panels show mesophyll cell walls. Arrows mark stromules. BF, bulliform cell; BS, outer bundle-sheath cell; C, chloroplast; CW, mesophyll cell wall; E, epidermis; IC, intercellular airspace; M, mesophyll cell; MI, mitochondria; N, nucleus; S, stoma; SC, sclerenchyma strand; VB, vascular bundle; V, vacuole.

Estimation of the transverse sections of leaves by light microscopy revealed a similar general leaf structure among the three species (Fig. 6 left panels). Significant variations in some anatomical traits were observed among species and among cultivars within the *O. sativa* species (Table 3). Wheat mesophyll tissue was about twice as thick as that of *O. sativa* cultivars and almost three times as thick as that of CG14 (Fig. 6). No strong drought effect was observed on *T*_m_ (Supplementary Table S1). The variation observed in the surface area of mesophyll cells exposed to the intercellular airspace per leaf area (*S*_m_) was mainly between rice and wheat (Table 3), with wheat having a higher *S*_m_ than all rice cultivars even when the curvature factor 1.25 was used for wheat (Supplementary Fig. S5B). *S*_m_ was significantly increased in wheat cultivars under drought (*P*<0.05), whereas no clear trend was observed in rice cultivars (Supplementary Fig. S5B).

In both rice and wheat, more than 60% of the exposed mesophyll cell surface area was covered by chloroplasts (*S*_c_) (Table 3). Wheat cultivars and upland rice cultivar Sal had the highest *S*_c_/*S*_m_ ratio, and CG14 had the lowest. A significant decrease in *S*_c_/*S*_m_ was observed in IR64 and aerobic rice cultivars, whereas an increase was recorded in CG14 under the SD condition (Supplementary Fig. S5C). No strong negative effect on *S*_c_/*S*_m_ was observed in upland rice and wheat cultivars under either drought condition (Supplementary Fig. S5C). *S*_c_ varied significantly among species and among rice cultivars, with Sal having a higher *S*_c_ value than other rice cultivars (Table 3). Wheat cultivars showed a clear increase in *S*_c_ under drought, whereas no clear *S*_c_ response was observed in rice cultivars (Supplementary Fig. S5D). A thinner mesophyll cell wall was observed in wheat cultivars than in all rice cultivars (Fig. 6), and drought increased *T*_w_, with the most significant effect observed in IR64, Sal and We4 under the SD condition (Supplementary Fig. S5E). *T*_w_ was also observed to be the only anatomical parameter to correlate with *N*_a_, a parameter related to leaf physiological maturity (Supplementary Fig. S6).

**Fig. 7.**
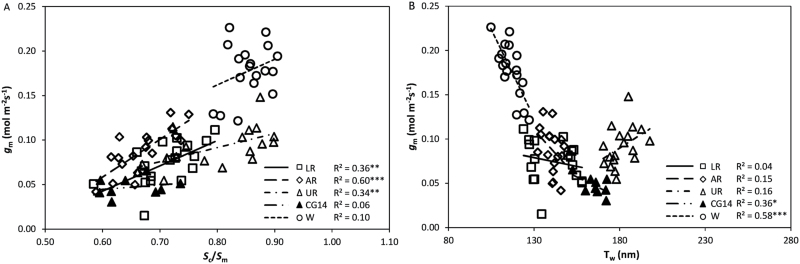
The relationships (A) between mesophyll conductance (*g*_m_) and the ratio of the exposed chloroplast surface area to the exposed surface area of mesophyll cell walls (*S*_c_/*S*_m_), and (B) between *g*_m_ and the thickness of the mesophyll cell wall (*T*_w_). AR, aerobic rice; CG14, *O. glaberrima*; LR, lowland rice; UR, upland rice; W, wheat. Linear regressions were fitted for each genotype group. The significance of each correlation is shown by asterisks: *P<0.05, **P<0.01, ***P<0.001.

Contributions of leaf anatomical properties to the variation in *g*_m_ were different in wheat and rice. A higher *g*_m_ in wheat was associated with a thicker *T*_m_, a higher *S*_m_, a higher *S*_c_ and a thinner *T*_w_ compared with rice cultivars, and these associations were little altered by using a different curvature factor for calculating *S*_m_ in wheat (Supplementary Fig. S7). However, no clear relationship was observed between *g*_m_ and *T*_m_ or between *g*_m_ and *S*_m_ within *O. sativa* or within most genotype groups (Supplementary Fig. S7A, B). *g*_m_ was positively correlated with *S*_c_ (Supplementary Fig. S7C; *R*^2^=0.14, *P*<0.01) and with *S*_c_/*S*_m_ (Fig. 7A; *R*^2^=0.33, *P*<0.001) among rice cultivars. *g*_m_ and *T*_w_ were negatively correlated among wheat cultivars (Fig. 7B), and this correlation only applied among rice cultivars if upland rice was excluded (*R*^2^=0.37, *P*<0.001). These correlations were also confirmed by multiple regression analysis, which indicated that *S*_c_/*S*_m_ contributed mostly to the variation in *g*_m_ within *O. sativa*, whereas *T*_w_ was the main determinant of *g*_m_ in wheat (Supplementary Table S4).

## Discussion

### Importance of *g*_s_ and *g*_m_ in determining *A*_n_ and TE

The photosynthetic capacity of leaves is closely related to their nitrogen content (Evans, 1989). In our study, the large variations in *A*_n_ cannot be explained by the small difference in leaf *N*_a_ among and within species (Supplementary Fig. S1B). The close correlation between *A*_n_ and total leaf conductance (*g*_tot_) among species as well as within each group (Fig. 2A) suggests that the variation in CO_2_ diffusion limitation could explain the genetic variation in *A*_n_. Further, *A*_n_ was correlated with CO_2_ diffusional conductance (Fig. 2B, C), especially with *g*_m_, in agreement with previous reports (Flexas *et al.*, 2008; Warren, 2008). It is of note that aerobic rice, upland rice, and wheat showed higher dependences of *A*_n_ on *g*_m_ than lowland rice (Supplementary Table S2).

TE was influenced mostly by *g*_s_ within each group (Supplementary Table S3); and this is not surprising given that *g*_s_ directly controls the transpirational water flow from leaf to the ambient air. However, *g*_m_ can influence *A*_n_ with no direct impact on transpiration and therefore can influence TE (Barbour *et al.*, 2010; Jahan *et al.*, 2014). For this reason, it has previously been suggested that the *g*_m_/*g*_s_ ratio is a potential target for improving TE under drought in several crops (Flexas *et al.*, 2010; Galmés *et al.*, 2011; Gu *et al.*, 2012; Flexas *et al.*, 2013) and that the ideal plants for arid environment have a high *g*_m_/*g*_s_ ratio (Condon *et al.*, 2004; Giuliani *et al.*, 2013). The observed relationship between TE and *g*_m_/*g*_s_ supports this (Fig. 2D). In the literature, a relationship between an intrinsic TE (*A*_n_/*g*_s_) and *g*_m_/*g*_s_ has often been shown to exclude any influence of VPD on the relationship, especially when data are collected from a wide range of sources (Flexas *et al.*, 2013). Mathematically, this is equivalent to the relationship between *A*_n_ and *g*_m_ (Fig. 2C). The somewhat more scattered relation in Fig. 2D than in Fig. 2C may reflect the influence of VPD, which varied between 1.0 and 1.6 kPa during our measurements. However, we found no systematic effect of such a small range of VPD on transpiration and TE (results not shown), and therefore we preferred to use TE for our illustration instead of intrinsic TE, which has a different biological unit and is more difficult to interpret in agronomic terms. In our case, a higher *g*_m_/*g*_s_ ratio in aerobic rice, upland rice, and wheat was found to be associated with higher TE, whereas lowland rice cultivars had a low *g*_m_/*g*_s_ ratio and low TE (Fig. 2D). Furthermore, there was a higher slope for the linear relationship in TE *versus* the *g*_m_/*g*_s_ ratio in upland rice, *O. glaberrima*, and wheat (Fig. 2D), indicating that the sensitivity of TE in response to the *g*_m_/*g*_s_ ratio was higher in drought-tolerant genotypes than in drought-susceptible genotypes.

Although *g*_m_ is expected to have little instantaneous impact on transpiration, our data showed that like *g*_s_, *g*_m_ decreased simultaneously under drought in all cultivars (Fig. 1B, C). This indicates that long-term drought during growth affected *g*_m_ in our experiment, in line with reports for several species (Miyazawa *et al.*, 2008; Flexas *et al.*, 2009), including rice (Gu *et al.*, 2012). When *g*_s_ and *g*_m_ had a rather parallel decrease, such as in lowland rice and We4 under the MD and the SD condition, or when *g*_m_ showed a stronger decrease than *g*_s_, such as in aerobic rice and UPL7 under the SD condition, no benefit for TE was observed. Only cultivars with a small decrease in *g*_m_ combined with a strong decrease in *g*_s_ under drought showed strong increases in TE (e.g. Sal, *O. glaberrima*, and S82). Therefore, a strong response of *g*_s_ combined with a small response of *g*_m_ would be beneficial for water conservation and increasing TE under long-term drought.

### Anatomical determinants of *g*_s_

Stomatal conductance (*g*_s_) is influenced by the structural traits and the opening of stomata (Xu and Zhou, 2008; Franks and Beerling, 2009; Galmés *et al.*, 2013). In our study, *D* or *S* alone could not explain the variation in *g*_s_ (Supplementary Fig. S4). *S* and *D* together determine the total pore area (stomatal area index, SAI) and the maximum stomatal conductance *g*_smax_ (Franks and Beerling, 2009; Galmés *et al.*, 2013). Our results confirmed this with marginally significant correlations (*P*<0.05) when *g*_s_ was plotted *versus* SAIs (Fig. 5A) or *versus g*_smax_ (Fig. 5B). The variation in SAIs or in *g*_smax_ within rice species was more associated with *D* than with *S*. Drought-tolerant types of aerobic rice, upland rice, and *O. glaberrima* had lower *D* than lowland rice (Table 3), in line with reports that mutants with reduced leaf stomatal density had enhanced the drought tolerance and WUE of plants (Yu *et al.*, 2008; Franks *et al.*, 2015). Furthermore, the difference in *D* was larger on the adaxial leaf surface than on the abaxial leaf surface, indicating that rice plants adapted to drier environments have developed fewer stomata on the upper surface to avoid direct water loss.

An increase in *D* under drought has been reported in several species (Martinez *et al.*, 2007; Hamanishi *et al.*, 2012), but this response has not been widely observed in other species (Xu and Zhou, 2008; Galmés *et al.*, 2013). On the other hand, a decrease in *S* in response to drought was consistent in those studies. In our study, cultivars with a smaller *S* under drought were accompanied by a greater reduction in *g*_s_ and *sg*_s_ (e.g. IR64, Sal, CG14, and S82). This is in line with the idea that smaller stomata are capable of a faster response to drought, compared with larger stomata (Franks and Beerling, 2009).

The stomatal properties examined in our study concerned only the anatomical features. However, biochemical components such as abscisic acid also influence *g*_s_, especially under drought (Franks and Farquhar, 2007). Such biochemical components influence the opening of stomata and may be responsible for part of the variability observed in *g*_s_.

### Anatomical determinants of *g*_m_

Leaf mesophyll thickness (*T*_m_) and *S*_m_ are the critical structural components that impact upon *g*_m_ (Evans *et al.*, 1994, 2009; Terashima *et al.*, 2011; Giuliani *et al.*, 2013). In our study, wheat cultivars had a thicker *T*_m_, a higher *S*_m_, and a higher *g*_m_ than rice cultivars. However, the difference in *T*_m_ or *S*_m_ was not associated with *g*_m_ variation within each species (Supplementary Fig. S7A, B). Drought had a stronger effect on *S*_m_ in wheat than in rice cultivars. This higher increase in *S*_m_ in wheat cultivars might contribute to the smaller decline in *g*_m_ under drought conditions (Galmés *et al.*, 2013).

It has been suggested that mesophyll cell walls account for more than 25% of total mesophyll resistance (Evans *et al.*, 2009; Terashima *et al.*, 2011; Tholen and Zhu, 2011). Han *et al.* (2016) reported that the decreasing *g*_m_ in cotton leaf under drought was associated with increased *T*_w_. Variation in *g*_m_ between *O. sativa* and wild rice species was suggested to be attributed to the thicker *T*_w_ in wild rice species (Scafaro *et al.*, 2011). In our study, wheat cultivars had a thinner *T*_w_ than all rice cultivars. However, *T*_w_ varied significantly among rice cultivars and the difference was not associated with observed differences in *g*_m_ (Fig. 7B). Increased *T*_w_ under drought was observed in our study, but this did not lead to a decreased *g*_m_, particularly in cultivars Sal and CG14. This suggests that *T*_w_ might not be the most dominant limiting factor for *g*_m_ under drought in rice. On the other hand, it has generally been proposed that thick mesophyll cell walls are beneficial for plant drought tolerance (Scafaro *et al.*, 2011; Giuliani *et al.*, 2013). The thicker *T*_w_ found in drought-tolerant cultivars (e.g. upland rice and *O. glaberrima*) than in drought-sensitive cultivars might support this idea.

*S*_c_ affects *g*_m_ in many species (Tholen *et al.*, 2008; Scafaro *et al.*, 2011; Terashima *et al.*, 2011; Tosens *et al.*, 2012*a*; Tomás *et al.*, 2013). Barbour *et al.* (2016) showed that the decrease in *g*_m_ in aged wheat and maize leaves was accompanied by a decline in *S*_c_/*S*_m_. In our study, the difference in *g*_m_ was explained somewhat more by *S*_c_/*S*_m_ (Fig. 7A) than by *S*_c_ (Supplementary Fig. S7D), particularly in rice cultivars. In response to drought, *g*_m_ decreased more in cultivars with clear decreased *S*_c_/*S*_m_ (e.g. lowland and aerobic rice) and decreased less in cultivars with a relatively constant *S*_c_/*S*_m_ ratio (e.g. upland rice and wheat) across water treatments. The increase in *S*_c_/*S*_m_ observed in *O. glaberrima* (Supplementary Fig. S5C) might be a compensating strategy for the increase in *T*_w_ under drought (Tosens *et al.*, 2012*a*), which resulted in a small decrease in *g*_m_. The high correlation between *g*_m_ and *S*_c_/*S*_m_ within rice (Fig. 7A) indicates that *S*_c_/*S*_m_ might be very important in determining *g*_m_ in rice.

It has been suggested that certain biochemical components such as aquaporins and carbonic anhydrase are *g*_m_-related (Miyazawa *et al.*, 2008; Flexas *et al.*, 2012). These characteristics were not investigated in our study. They might explain the part of the variability observed in *g*_m_ that was not explained by anatomical features.

## Conclusions

Stomatal conductance and mesophyll conductance explained most of the variability in *A*_n_ among species and within the species *O. sativa*. The higher *g*_m_/*g*_s_ ratio in aerobic rice, upland rice, and wheat cultivars was associated with higher TE, compared with lowland rice cultivars and *O. glaberrima*. Our study revealed the genotypic variation in the TE *versus g*_m_/*g*_s_ relationship among species and within the species *O. sativa*. There was a higher TE sensitivity in response to *g*_m_/*g*_s_ in drought-tolerant groups (*O. glaberrima*, upland rice, and wheat) than in lowland and aerobic rice. In particular, upland rice, *O. glaberrima*, and wheat cultivars minimized the decrease in *g*_m_; this can simultaneously sustain photosynthesis and increase TE under drought.

Stomatal development was responsible for the variation in *g*_s_ among contrasting rice types and between rice and wheat. SAIs and *g*_smax_ were correlated with the genetic variation of *g*_s_ among three species. More specifically, stomatal density (one component affecting SAIs and *g*_smax_) was more related to the genetic variation in *g*_s_ among rice cultivars, suggesting that drought-tolerant rice cultivars might have developed a mechanism of stomatal acclimation to drier edaphic environments. Moreover, cultivars with smaller stomata had a stronger decrease in *g*_s_, and thus a higher increase in TE, under drought stress.

Previous studies indicated that thick *T*_m_, high *S*_m_, high *S*_c_, and thin *T*_w_ were associated with high *g*_m_. These correlations were observed between wheat and rice but not among rice cultivars in our study. More importantly, for rice cultivars, the adverse impact of thick *T*_w_ can be neutralized by other anatomical factors such as a high *S*_c_/*S*_m_ ratio. In particular, under drought, the maintained or increased *S*_c_/*S*_m_ ratio in upland rice, *O. glaberrima*, and wheat cultivars resulted in a smaller decrease in *g*_m_ in them than in lowland and aerobic rice cultivars.

In short, our results suggest that rice TE might be improved by modulating stomatal and mesophyll structural traits via breeding selection and/or genetic engineering.

## Supplementary data

Supplementary data are available at JXB online.

Fig. S1. Response of leaf nitrogen per unit area (*N*_a_) of rice and wheat cultivars to water treatments, relationship between photosynthetic rate (*A*_n_) and *N*_a_, and relationship between mesophyll conductance (*g*_m_) and *N*_a_.

Fig. S2. Relationship between transpiration efficiency (TE) and stomatal conductance (*g*_s_), and between TE and mesophyll conductance (*g*_m_).

Fig. S3. Stomatal density (*D*) and size (*S*) from adaxial side and abaxial side of rice and wheat cultivars under three treatments.

Fig. S4. The relationships between stomatal conductance (*g*_s_) and stomatal density (*D*), and between *g*_s_ and stomatal size (*S*).

Fig. S5. Mesophyll cell properties of wheat and rice leaves obtained from light and electron microscope images under three treatments.

Fig. S6. Relationships between mesophyll thickness (*T*_m_) and *N*_a_, the surface area of mesophyll cells exposed to the intercellular airspaces per leaf area (*S*_m_) and *N*_a_, ratio of the exposed surface area of chloroplast to the exposed surface area of mesophyll cell walls (*S*_c_/*S*_m_) and *N*_a_, the surface area of chloroplasts exposed to intercellular airspace per leaf area (*S*_c_) and *N*_a_, and thickness of the mesophyll cell wall (*T*_w_) and *N*_a_.

Fig. S7. The relationship between mesophyll conductance (*g*_m_) and mesophyll thickness (*T*_m_), the surface area of mesophyll cells exposed to the intercellular airspaces per leaf area (*S*_m_), and the surface area of chloroplasts exposed to intercellular airspace per leaf area (*S*_c_).

Table S1. A two-way analysis of variance of genotype *versus* water stress for measured and estimated photosynthetic and anatomical parameters.

Table S2. Multiple regression analysis of light-saturated photosynthesis (*A*_n_) as a function of *g*_s_ and *g*_m_, based on data of three treatments.

Table S3. Multiple regression analysis of transpiration efficiency (TE) as a function of *g*_s_ and *g*_m_, based on data of three treatments.

Table S4. Multiple regression analysis of mesophyll conductance (*g*_m_) as a function of *T*_w_, *S*_c_/*S*_m_ and *N*_a_, based on combined data of all three water treatments.

## Supplementary Material

Supplementary_Table_S1_S4_Fig_S1-S7

## References

[CIT0001] Abràmoff MD Magalhães PJ Ram SJ 2004 Image processing with ImageJ. Biophotonics International 11, 36–42.

[CIT0002] Atlin G Lafitte H Tao D Laza M Amante M Courtois B 2006 Developing rice cultivars for high-fertility upland systems in the Asian tropics. Field Crops Research 97, 43–52.

[CIT0003] Barbour MM Evans JR Simonin KA von Caemmerer S 2016 Online CO_2_ and H_2_O oxygen isotope fractionation allows estimation of mesophyll conductance in C_4_ plants, and reveals that mesophyll conductance decreases as leaves age in both C_4_ and C_3_ plants. New Phytologist 210, 875–889. 26778088 10.1111/nph.13830

[CIT0004] Barbour MM Warren CR Farquhar GD Forrester G Brown H 2010 Variability in mesophyll conductance between barley genotypes, and effects on transpiration efficiency and carbon isotope discrimination. Plant, Cell & Environment 33, 1176–1185. 10.1111/j.1365-3040.2010.02138.x 20199618

[CIT0005] Bouman B Peng S Castaneda A Visperas R 2005 Yield and water use of irrigated tropical aerobic rice systems. Agricultural Water Management 74, 87–105.

[CIT0006] Bouman B Tuong TP 2001 Field water management to save water and increase its productivity in irrigated lowland rice. Agricultural Water Management 49, 11–30.

[CIT0007] Chaves MM Pereira JS Maroco J Rodrigues ML Ricardo CP Osório ML Carvalho I Faria T Pinheiro C 2002 How plants cope with water stress in the field. Photosynthesis and growth. Annals of Botany 89, 907–916. 12102516 10.1093/aob/mcf105 PMC4233809

[CIT0008] Chonan N 1970 Studies on the photosynthetic tissues in the leaves of cereal crops. V. Comparison of the mesophyll structure among seedling leaves of cereal crops. Crop Science Society of Japan 39, 418–425.

[CIT0009] Chonan N 1972 Differences in mesophyll structures between temperate and tropical grasses. Crop Science Society of Japan 41, 414–419.

[CIT0010] Condon AG Richards RA Rebetzke GJ Farquhar GD 2004 Breeding for high water-use efficiency. Journal of Experimental Botany 55, 2447–2460. 15475373 10.1093/jxb/erh277

[CIT0011] Evans JR 1989 Photosynthesis and nitrogen relationships in leaves of C_3_ plants. Oecologia 78, 9–19. 28311896 10.1007/BF00377192

[CIT0012] Evans JR Kaldenhoff R Genty B Terashima I 2009 Resistances along the CO_2_ diffusion pathway inside leaves. Journal of Experimental Botany 60, 2235–2248. 19395390 10.1093/jxb/erp117

[CIT0013] Evans JR Vellen L 1996 Wheat cultivars differ in transpiration efficiency and CO_2_ diffusion inside their leaves. In: Ishii R Horie T, eds. Crop research in Asia: achievements and perspective. Fukui, Japan: Asian Crop Science Association, 326–329.

[CIT0014] Evans JR von Caemmerer S Setchell BA Hudson GS 1994 The relationship between CO_2_ transfer conductance and leaf anatomy in transgenic tobacco with a reduced content of Rubisco. Functional Plant Biology 21, 475–495.

[CIT0015] Flexas J Barbour MM Brendel O Cabrera HM Carriquí M Díaz-Espejo A Douthe C Dreyer E Ferrio JP Gago J 2012 Mesophyll diffusion conductance to CO_2_: an unappreciated central player in photosynthesis. Plant Science 193, 70–84. 22794920 10.1016/j.plantsci.2012.05.009

[CIT0016] Flexas J Barón M Bota J 2009 Photosynthesis limitations during water stress acclimation and recovery in the drought-adapted *Vitis* hybrid Richter-110 (*V. berlandieri×V. rupestris*). Journal of Experimental Botany 60, 2361–2377. 19351904 10.1093/jxb/erp069

[CIT0017] Flexas J Díaz-Espejo A Berry JA Cifre J Galmés J Kaldenhoff R Medrano H Ribas-Carbó M 2007 Analysis of leakage in IRGA’s leaf chambers of open gas exchange systems: quantification and its effects in photosynthesis parameterization. Journal of Experimental Botany 58, 1533–1543. 17339650 10.1093/jxb/erm027

[CIT0018] Flexas J Galmés J Gallé A Gulías J Pou A Ribas-Carbó M Tomàs M Medrano H 2010 Improving water use efficiency in grapevines: potential physiological targets for biotechnological improvement. Australian Journal of Grape and Wine Research 16, 106–121.

[CIT0019] Flexas J Niinemets U Gallé A 2013 Diffusional conductances to CO_2_ as a target for increasing photosynthesis and photosynthetic water-use efficiency. Photosynthesis Research 117, 45–59. 23670217 10.1007/s11120-013-9844-z

[CIT0020] Flexas J Ribas-Carbó M Diaz-Espejo A Galmés J Medrano H 2008 Mesophyll conductance to CO_2_: current knowledge and future prospects. Plant, Cell & Environment 31, 602–621. 10.1111/j.1365-3040.2007.01757.x 17996013

[CIT0021] Franks PJ Farquhar GD 2007 The mechanical diversity of stomata and its significance in gas-exchange control. Plant Physiology 143, 78–87. 17114276 10.1104/pp.106.089367 PMC1761988

[CIT0022] Franks PJ Beerling DJ 2009 Maximum leaf conductance driven by CO_2_ effects on stomatal size and density over geologic time. Proceedings of the National Academy of Sciences, USA 106, 10343–10347. 10.1073/pnas.0904209106 PMC2693183 19506250

[CIT0023] Franks PJ W Doheny-Adams T Britton-Harper ZJ Gray JE 2015 Increasing water-use efficiency directly through genetic manipulation of stomatal density. New Phytologist 207, 188–195. 25754246 10.1111/nph.13347

[CIT0024] Galmés J Conesa MÀ Ochogavía JM Perdomo JA Francis DM Ribas-Carbó M Savé R Flexas J Medrano H Cifre J 2011 Physiological and morphological adaptations in relation to water use efficiency in Mediterranean accessions of *Solanum lycopersicum*. Plant, Cell & Environment 34, 245–260. 10.1111/j.1365-3040.2010.02239.x 20955222

[CIT0025] Galmés J Ochogavía JM Gago J Roldán EJ Cifre J Conesa MÀ 2013 Leaf responses to drought stress in Mediterranean accessions of *Solanum lycopersicum*: anatomical adaptations in relation to gas exchange parameters. Plant, Cell & Environment 36, 920–935. 10.1111/pce.12022 23057729

[CIT0026] Genty B Briantais J-M Baker NR 1989 The relationship between the quantum yield of photosynthetic electron transport and quenching of chlorophyll fluorescence. Biochimica et Biophysica Acta 990, 87–92.

[CIT0027] George T Magbanua R Garrity DP Tubana BS Quiton J 2002 Rapid yield loss of rice cropped successively in aerobic soil. Agronomy Journal 94, 981–989.

[CIT0028] Giday H Kjaer KH Fanourakis D Ottosen CO 2013 Smaller stomata require less severe leaf drying to close: a case study in *Rosa hydrida*. Journal of Plant Physiology 170, 1309–1316. 23726470 10.1016/j.jplph.2013.04.007

[CIT0029] Giuliani R Koteyeva N Voznesenskaya E Evans MA Cousins AB Edwards GE 2013 Coordination of leaf photosynthesis, transpiration, and structural traits in rice and wild relatives (genus *Oryza*). Plant Physiology 162, 1632–1651. 23669746 10.1104/pp.113.217497 PMC3707562

[CIT0030] Gorton HL Herbert SK Vogelmann TC 2003 Photoacoustic analysis indicates that chloroplast movement does not alter liquid-phase CO_2_ diffusion in leaves of *Alocasia brisbanensis*. Plant Physiology 132, 1529–1539. 12857833 10.1104/pp.102.019612 PMC167091

[CIT0031] Gu J Yin X Stomph TJ Wang H Struik PC 2012 Physiological basis of genetic variation in leaf photosynthesis among rice (*Oryza sativa* L.) introgression lines under drought and well-watered conditions. Journal of Experimental Botany 63, 5137–5153. 22888131 10.1093/jxb/ers170 PMC3430991

[CIT0032] Haefele S Siopongco J Boling A Bouman B Tuong T 2009 Transpiration efficiency of rice (*Oryza sativa* L.). Field Crops Research 111, 1–10.

[CIT0033] Hamanishi ET Thomas BR Campbell MM 2012 Drought induces alterations in the stomatal development program in *Populus*. Journal of Experimental Botany 63, 4959–4971. 22760471 10.1093/jxb/ers177 PMC3427991

[CIT0034] Han JM Meng HF Wang SY Jiang CD Liu F Zhang WF Zhang YL 2016 Variability of mesophyll conductance and its relationship with water use efficiency in cotton leaves under drought pretreatment. Journal of Plant Physiology 194, 61–71. 27101723 10.1016/j.jplph.2016.03.014

[CIT0035] Jahan E Amthor JS Farquhar GD Trethowan R Barbour MM 2014 Variation in mesophyll conductance among Australian wheat genotypes. Functional Plant Biology 41, 568–580. 10.1071/FP13254 32481014

[CIT0036] Kadam NN Yin X Bindraban PS Struik PC Jagadish KS 2015 Does morphological and anatomical plasticity during the vegetative stage make wheat more tolerant of water deficit stress than rice?Plant Physiology 167, 1389–1401. 25614066 10.1104/pp.114.253328 PMC4378155

[CIT0037] Kemanian AR Stöckle CO Huggins DR 2005 Transpiration-use efficiency of barley. Agricultural and Forest Meteorology 130, 1–11.

[CIT0038] Lawlor DW Tezara W 2009 Causes of decreased photosynthetic rate and metabolic capacity in water-deficient leaf cells: a critical evaluation of mechanisms and integration of processes. Annals of Botany 103, 561–579. 19155221 10.1093/aob/mcn244 PMC2707350

[CIT0039] Liu J Liao D Oane R Estenor L Yang X Li Z Bennett J 2006 Genetic variation in the sensitivity of anther dehiscence to drought stress in rice. Field Crops Research 97, 87–100.

[CIT0040] Loriaux SD Avenson TJ Welles JM McDermitt DK Eckles RD Riensche B Genty B 2013 Closing in on maximum yield of chlorophyll fluorescence using a single multiphase flash of sub-saturating intensity. Plant, Cell & Environment 36, 1755–1770. 10.1111/pce.12115 23586649

[CIT0041] Makino A Mae T Ohira K 1988 Differences between wheat and rice in the enzymic properties of ribulose-1,5-bisphosphate carboxylase/oxygenase and the relationship to photosynthetic gas exchange. Planta 174, 30–38. 24221414 10.1007/BF00394870

[CIT0042] Martinez J Silva H Ledent J Pinto M 2007 Effect of drought stress on the osmotic adjustment, cell wall elasticity and cell volume of six cultivars of common beans (*Phaseolus vulgaris* L.). European Journal of Agronomy 26, 30–38.

[CIT0043] Miyazawa S-I Yoshimura S Shinzaki Y Maeshima M Miyake C 2008 Deactivation of aquaporins decreases internal conductance to CO_2_ diffusion in tobacco leaves grown under long-term drought. Functional Plant Biology 35, 553–564. 10.1071/FP08117 32688811

[CIT0044] Nuijten E van Treuren R Struik PC Mokuwa A Okry F Teeken B Richards P 2009 Evidence for the emergence of new rice types of interspecific hybrid origin in West African farmers’ fields. PLoS ONE 4, e7335. 19806197 10.1371/journal.pone.0007335 PMC2752159

[CIT0045] Ohsumi A Kanemura T Homma K Horie T Shiraiwa T 2007 Genotypic variation of stomatal conductance in relation to stomatal density and length in rice (*Oryza sativa* L.). Plant Production Science 10, 322–328.

[CIT0046] Pinheiro C Chaves MM 2011 Photosynthesis and drought: can we make metabolic connections from available data?Journal of Experimental Botany 62, 869–882. 21172816 10.1093/jxb/erq340

[CIT0047] Praba ML Cairns J Babu R Lafitte H 2009 Identification of physiological traits underlying cultivar differences in drought tolerance in rice and wheat. Journal of Agronomy and Crop Science 195, 30–46.

[CIT0048] Sage TL Sage RF 2009 The functional anatomy of rice leaves: implications for refixation of photorespiratory CO_2_ and efforts to engineer C_4_ photosynthesis into rice. Plant & Cell Physiology 50, 756–772. 19246459 10.1093/pcp/pcp033

[CIT0049] Sarla N Swamy BM 2005 *Oryza glaberrima*: a source for the improvement of *Oryza sativa*. Current Science Bangalore 89, 955–963.

[CIT0050] Scafaro AP Von Caemmerer S Evans JR Atwell BJ 2011 Temperature response of mesophyll conductance in cultivated and wild *Oryza* species with contrasting mesophyll cell wall thickness. Plant, Cell & Environment 34, 1999–2008. 10.1111/j.1365-3040.2011.02398.x 21752031

[CIT0051] Scartazza A Lauteri M Guido M Brugnoli E 1998 Carbon isotope discrimination in leaf and stem sugars, water-use efficiency and mesophyll conductance during different developmental stages in rice subjected to drought. Functional Plant Biology 25, 489–498.

[CIT0052] Singh S Ladha J Gupta R Bhushan L Rao A 2008 Weed management in aerobic rice systems under varying establishment methods. Crop Protection 27, 660–671.

[CIT0053] Smith S Weyers J Berry W 1989 Variation in stomatal characteristics over the lower surface of *Commelina communis* leaves. Plant, Cell & Environment 12, 653–659.

[CIT0054] Terashima I Hanba YT Tholen D Niinemets Ü 2011 Leaf functional anatomy in relation to photosynthesis. Plant Physiology 155, 108–116. 21075960 10.1104/pp.110.165472 PMC3075775

[CIT0055] Thain J 1983 Curvature correction factors in the measurement of cell surface areas in plant tissues. Journal of Experimental Botany 34, 87–94.

[CIT0056] Thanh N Thanh N Zheng H Dong N Trinh L Ali M Nguyen H 1999 Genetic variation in root morphology and microsatellite DNA loci in upland rice (*Oryza sativa* L.) from Vietnam. Euphytica 105, 53–62.

[CIT0057] Tholen D Boom C Noguchi K Ueda S Katase T Terashima I 2008 The chloroplast avoidance response decreases internal conductance to CO_2_ diffusion in *Arabidopsis thaliana* leaves. Plant, Cell & Environment 31, 1688–1700. 10.1111/j.1365-3040.2008.01875.x 18721264

[CIT0058] Tholen D Zhu XG 2011 The mechanistic basis of internal conductance: a theoretical analysis of mesophyll cell photosynthesis and CO_2_ diffusion. Plant Physiology 156, 90–105. 21441385 10.1104/pp.111.172346 PMC3091052

[CIT0059] Tomás M Flexas J Copolovici L Galmés J Hallik L Medrano H Ribas-Carbó M Tosens T Vislap V Niinemets Ü 2013 Importance of leaf anatomy in determining mesophyll diffusion conductance to CO_2_ across species: quantitative limitations and scaling up by models. Journal of Experimental Botany 64, 2269–2281. 23564954 10.1093/jxb/ert086 PMC3654418

[CIT0060] Tosens T Niinemets U Vislap V Eichelmann H Castro Díez P 2012 *a* Developmental changes in mesophyll diffusion conductance and photosynthetic capacity under different light and water availabilities in *Populus tremula*: how structure constrains function. Plant, Cell & Environment 35, 839–856. 10.1111/j.1365-3040.2011.02457.x 22070625

[CIT0061] Tosens T Niinemets Ü Westoby M Wright IJ 2012 *b* Anatomical basis of variation in mesophyll resistance in eastern Australian sclerophylls: news of a long and winding path. Journal of Experimental Botany 63, 5105–5119. 22888123 10.1093/jxb/ers171 PMC3430992

[CIT0062] Warren CR 2008 Stand aside stomata, another actor deserves centre stage: the forgotten role of the internal conductance to CO_2_ transfer. Journal of Experimental Botany 59, 1475–1487. 17975206 10.1093/jxb/erm245

[CIT0063] Xu Z Zhou G 2008 Responses of leaf stomatal density to water status and its relationship with photosynthesis in a grass. Journal of Experimental Botany 59, 3317–3325. 18648104 10.1093/jxb/ern185 PMC2529243

[CIT0064] Yang J Zhang J 2006 Grain filling of cereals under soil drying. New Phytologist 169, 223–236. 16411926 10.1111/j.1469-8137.2005.01597.x

[CIT0065] Yang J Zhang J Wang Z Liu L Zhu Q 2003 Postanthesis water deficits enhance grain filling in two-line hybrid rice. Crop Science 43, 2099–2108.

[CIT0066] Yin X Struik PC 2009 Theoretical reconsiderations when estimating the mesophyll conductance to CO_2_ diffusion in leaves of C_3_ plants by analysis of combined gas exchange and chlorophyll fluorescence measurements. Plant, Cell & Environment 32, 1513–1524 (corrigendum **33**, 1595). 10.1111/j.1365-3040.2009.02016.x 19558403

[CIT0067] Yin X Struik PC Romero P Harbinson J Evers JB Van der Putten PE Vos J 2009 Using combined measurements of gas exchange and chlorophyll fluorescence to estimate parameters of a biochemical C_3_ photosynthesis model: a critical appraisal and a new integrated approach applied to leaves in a wheat (*Triticum aestivum*) canopy. Plant, Cell & Environment 32, 448–464. 10.1111/j.1365-3040.2009.01934.x 19183300

[CIT0068] Yu H Chen X Hong YY Wang Y Xu P Ke SD Liu HY Zhu JK Oliver DJ Xiang CB 2008 Activated expression of an *Arabidopsis* HD-START protein confers drought tolerance with improved root system and reduced stomatal density. The Plant Cell 20, 1134–1151. 18451323 10.1105/tpc.108.058263 PMC2390749

[CIT0069] Zhang H Zhang S Yang J Zhang J Wang Z 2008 Postanthesis moderate wetting drying improves both quality and quantity of rice yield. Agronomy Journal 100, 726–734.

