# Supplementary material for: Stomatal conductance, mesophyll conductance, and transpiration efficiency in relation to leaf anatomy in rice and wheat genotypes under drought
Source: J Exp Bot. 2017 Sep 15;68(18):5191–205. doi: 10.1093/jxb/erx314 (PMC5853379; doi:10.1093/jxb/erx314)
Supplement: Supplementary_Table_S1_S4_Fig_S1-S7 [file erx314_suppl_supplementary_table_s1_s4_fig_s1-s7.pdf]

Supplementary tables

Table S1. A two-way analysis of variance of genotype versus water stress for measured and estimated photosynthetic and anatomical parameters.

Species		A_n	g_s	g_m	TE	T_m	S_m	S_c/S_m	S_c	T_w	$S_{adaxial}$	$D_{adaxial}$	$S_{abaxial}$	$D_{abaxial}$	$SAI_{adaxial}$	$SAI_{abaxial}$	SAIs	g_{smax}
	Cultivar	***	***	***	***	***	**	***	***	***	***	***	***	**	***	***	***	***
O. sativa	Treatment	*	***	***	**	ns	ns	*	ns	***	***	*	***	ns	ns	***	***	*
	C×T	ns	ns	ns	ns	ns	ns	ns	ns	ns	ns	ns	ns	ns	*	ns	*	ns
O. glaberrima	Cultivar																	
	Treatment	ns	*	ns	*	*	ns	*	ns	ns	*	ns	*	ns	ns	ns	ns	ns
	C×T																	
Lowland rice	Cultivar	ns	ns	ns	ns	***	ns	*	ns	***	***	***	***	**	ns	ns	ns	ns
	Treatment	*	**	***	*	ns	ns	ns	ns	*	*	*	***	ns	ns	**	*	ns
	C×T	ns	ns	ns	ns	ns	ns	ns	ns	ns	ns	ns	ns	ns	ns	ns	*	ns
Aerobic rice	Cultivar	*	ns	*	ns	**	ns	ns	ns	ns	ns	***	ns	ns	***	ns	**	***
	Treatment	ns	ns	*	ns	*	ns	*	ns	ns	*	ns	*	ns	ns	ns	ns	*
	C×T	ns	ns	ns	ns	ns	ns	ns	ns	ns	ns	ns	ns	*	**	ns	*	*
Upland rice	Cultivar	**	ns	**	**	***	*	***	***	***	ns	*	*	ns	ns	*	*	***
	Treatment	ns	*	*	ns	ns	ns	ns	*	*	ns	ns	*	ns	*	**	**	*
	C×T	ns	ns	ns	ns	ns	ns	ns	ns	ns	ns	ns	ns	ns	ns	ns	ns	ns
Wheat	Cultivar	ns	ns	*	ns	***	ns	ns	ns	***	ns	***	ns	***	*	***	***	***
	Treatment	ns	**	**	*	ns	*	ns	*	*	*	***	**	ns	ns	**	ns	ns
	C×T	ns	ns	ns	ns	ns	ns	ns	ns	ns	ns	ns	ns	ns	ns	*	*	*
Rice + Wheat	Cultivar	***	***	***	***	***	***	***	***	***	***	***	***	***	***	***	***	***
	Treatment	**	***	***	***	*	*	ns	ns	***	***	**	***	ns	ns	***	**	ns
	C×T	ns	ns	ns	ns	ns	ns	*	*	ns	**	ns	***	*	**	**	**	*

A_n = light-saturated photosynthesis; g_s = stomatal conductance; g_m = mesophyll conductance; TE = leaf transpiration efficiency; T_m = mesophyll thickness; S_m = mesophyll surface area exposed to intercellular airspace per leaf area; S_c/S_m = ratio of the exposed surface area of chloroplast to the exposed surface area of mesophyll cell walls; S_c = chloroplast surface area exposed to intercellular airspace per leaf area; T_w = mesophyll cell wall thickness; $S_{adaxial}$ ($S_{abaxial}$)= stomatal size on the adaxial (abaxial) leaf surface; $D_{adaxial}$ ($D_{abaxial}$) = stomatal density on the adaxial (abaxial) leaf surface; $SAI_{adaxial}$ ($SAI_{abaxial}$) = stomatal area index on the adaxial (abaxial) leaf surface; SAIs = summed stomatal area index on both leaf surfaces; g_{smax} = maximum stomatal conductance. The significance of each correlation: *, P< 0.05; **, P< 0.01; ***, P<0.001; ns, not significant.

Table S2. Multiple regression analysis of light-saturated photosynthesis (A_n) as a function of g_s and g_m ($A_n = b_0 + b_1 g_s + b_2 g_m$), based on data of three treatments.

Genotype	Intercept	Regression coefficient			
	b_0	b_1	b_2	R^2	No. of data points
Lowland rice	4.79	20.91**	23.83	0.85	18
Aerobic rice	6.58	16.13	25.30*	0.42	18
Upland rice	4.67	19.26*	59.76***	0.79	18
O. glaberrima	7.40	-19.69	50.02	0.52	9
O. sativa	5.55	9.10**	52.80***	0.59	54
Wheat	5.31	13.41	65.13***	0.79	18
Rice + Wheat	3.43	9.58**	77.76***	0.90	81

*,**,*** significant at the 0.05, 0.01 and 0.001 probability levels, respectively.

Table S3. Multiple regression analysis of TE as a function of g_s and g_m (TE = $b_0 + b_1 g_s + b_2 g_m$), based on data of three treatments.

Genotype	Intercept	Regression coefficient			
	b_0	b_1	b_2	R^2	No. of data points
Lowland rice	3.41	-4.19*	-0.48	0.69	18
Aerobic rice	5.46	-18.45*	8.03	0.34	18
Upland rice	4.78	-16.95***	19.01***	0.74	18
O. glaberrima	5.47	-27.06**	13.60	0.82	9
O. sativa	4.13	-12.11***	15.33***	0.72	54
Wheat	5.46	-10.38***	5.12	0.63	18
Rice + Wheat	4.07	-12.21***	15.33***	0.69	81

*,**,*** significant at the 0.05, 0.01 and 0.001 probability levels, respectively.

Table S4. Multiple regression analysis of mesophyll conductance (g_m) as a function of T_w, S_c/S_m and N_a ($g_m = b_0 + b_1T_w + b_2S_c/S_m + b_3N_a$), based on combined data of all three water treatments.

Genotype	Intercept	Regression coefficient				
	b_0	b_1	b_2	b_3	R^2	No. of data points
Lowland rice	-0.11	-0.03	0.33*	-0.04	0.43	18
Aerobic rice	-0.15	-0.18	0.42**	-0.02	0.60	18
Upland rice	-0.15	0.13	0.15	0.11	0.44	18
O. glaberrima	-0.06	0.37	0.24*	-0.17**	0.86	9
O. sativa	0.08	-0.67**	0.24***	-0.06**	0.40	54
Wheat	0.36	-4.04***	0.29	0.04	0.74	18
Rice + Wheat	0.04	-1.15***	0.36***	-0.05**	0.78	81

Supplementary figures

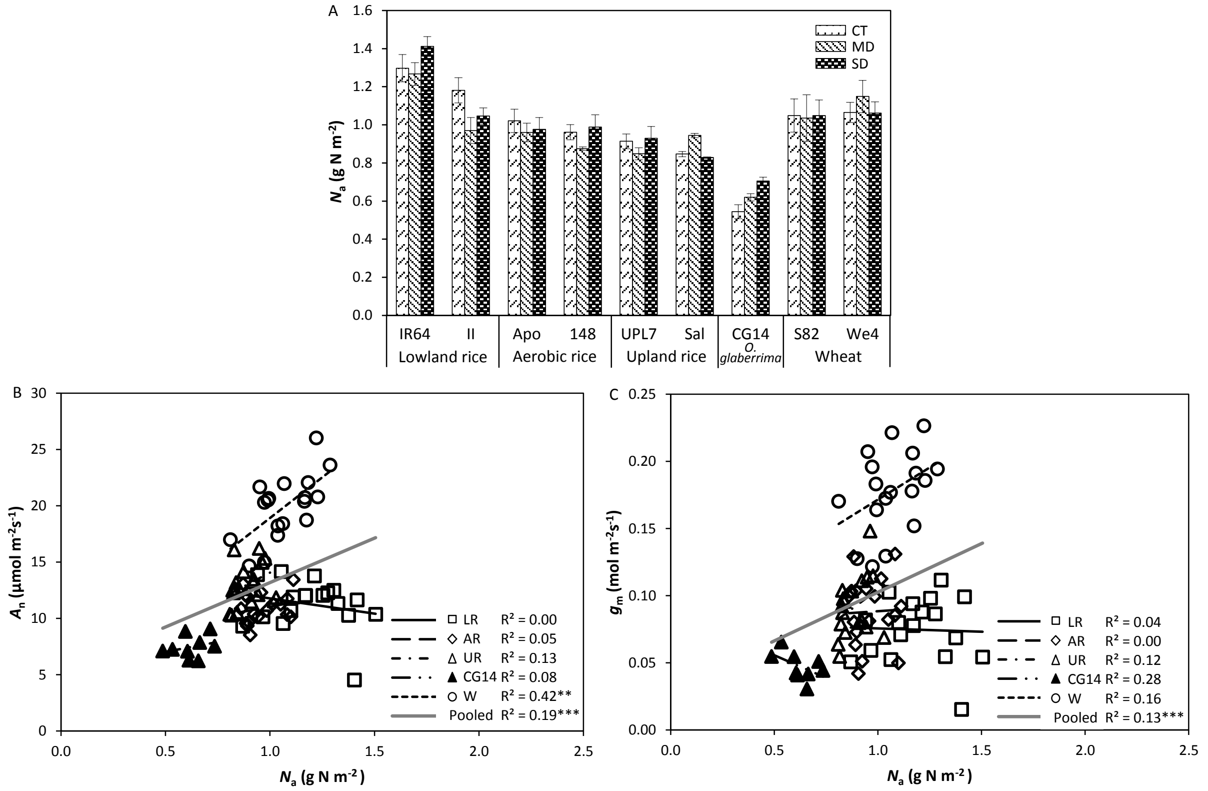

Fig. S1. (A) Response of leaf nitrogen per unit area (N_a) of rice and wheat cultivars to water stress treatments: control (CT); mild drought (MD); more severe drought (SD). (B) Relationship between photosynthetic rate (A_n) (400 µmol mol^{-1} CO_2, 1000-1500 µmol m^{-2} s^{-1} irradiance, and 25°C) and N_a; (C) Relationship between mesophyll conductance (g_m) and N_a. LR: lowland rice; AR: aerobic rice; UR: upland rice; CG14: *O. glaberrima*; W: wheat. Linear regressions were fitted for overall data and for each genotype group. The significance of each correlation is shown as: *, P< 0.05; **, P< 0.01; ***, P<0.001.

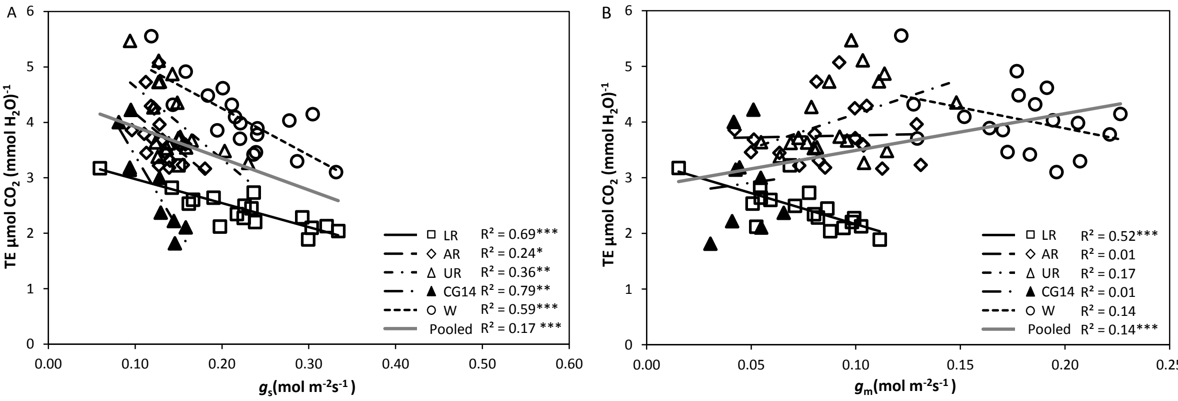

Fig. S2. Relationship between transpiration efficiency (TE) and stomatal conductance (g_s), and between TE and mesophyll conductance (g_m). Values of TE and g_s were obtained and calculated under 400 µmol mol^{-1} CO_2, 1000-1500 µmol m^{-2} s^{-1} irradiance, and 25℃. g_m was calculated based on the non-rectangular hyperbolic method (Yin and Struik, 2009). LR: lowland rice; AR: aerobic rice; UR: upland rice; CG14: *O. glaberrima*; W: wheat. Linear regressions were fitted for overall data and for each genotype group. The significance of each correlation is shown as: *, P< 0.05; **, P< 0.01; ***, P<0.001.

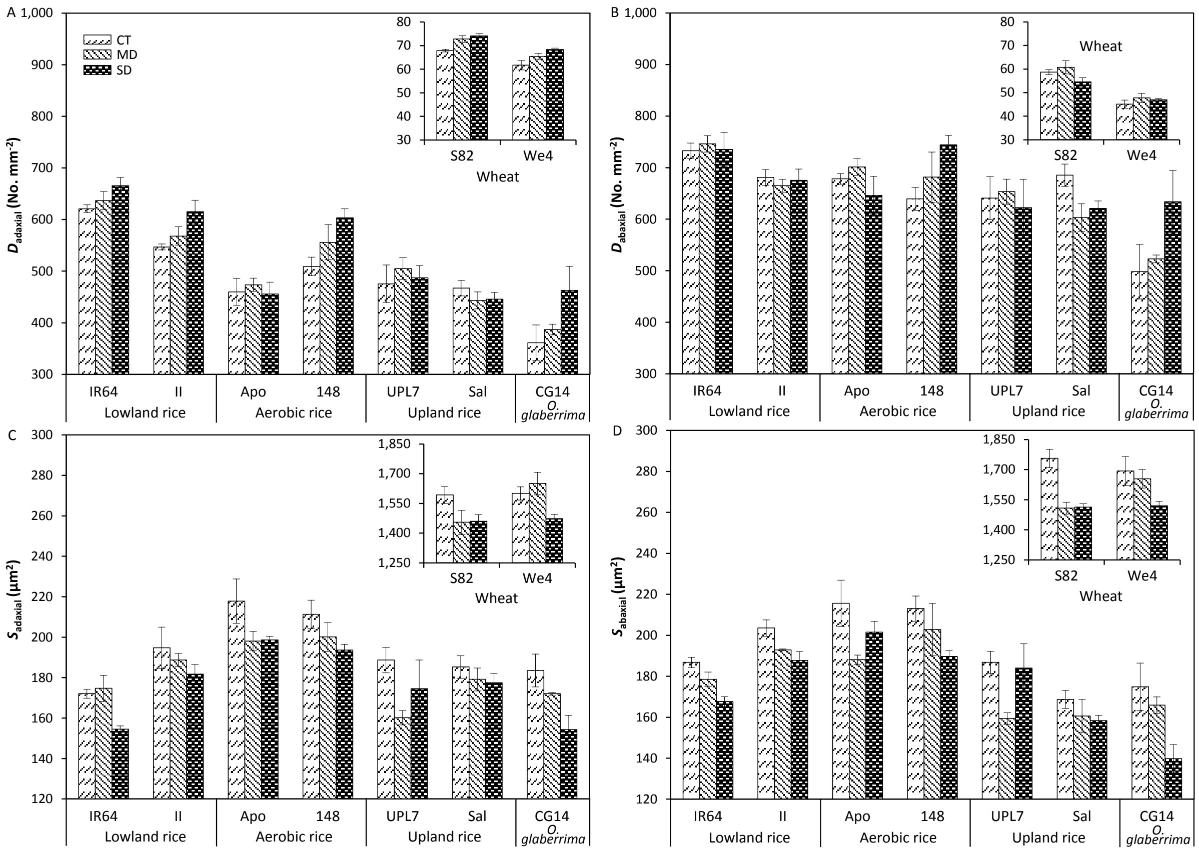

Fig. S3. Stomatal density (*D*) and size (*S*) from adaxial side (A, C) and abaxial side (B, D) of rice and wheat cultivars under three treatments: control (CT); mild drought (MD); and more severe drought (SD).

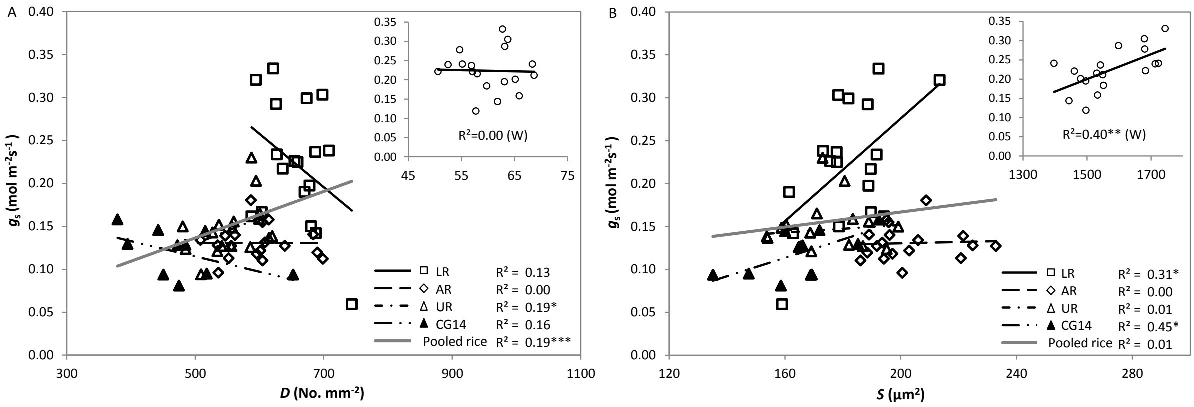

Fig. S4. The relationships between stomatal conductance (g_s) and stomatal density (*D*), and between g_s and stomatal size (*S*). Values of g_s were obtained and calculated under 400 µmol mol^{-1} CO_2, 1000-1500 µmol m^{-2} s^{-1} irradiance, and 25°C. *S* and *D* have been calculated as the average between both sides of the leaf. LR: lowland rice; AR: aerobic rice; UR: upland rice; CG14: *O. glaberrima*; W: wheat. Linear regressions were fitted for overall data and for each genotype group. The significance of each correlation is shown as: *, P< 0.05; **, P< 0.01; ***, P<0.001.

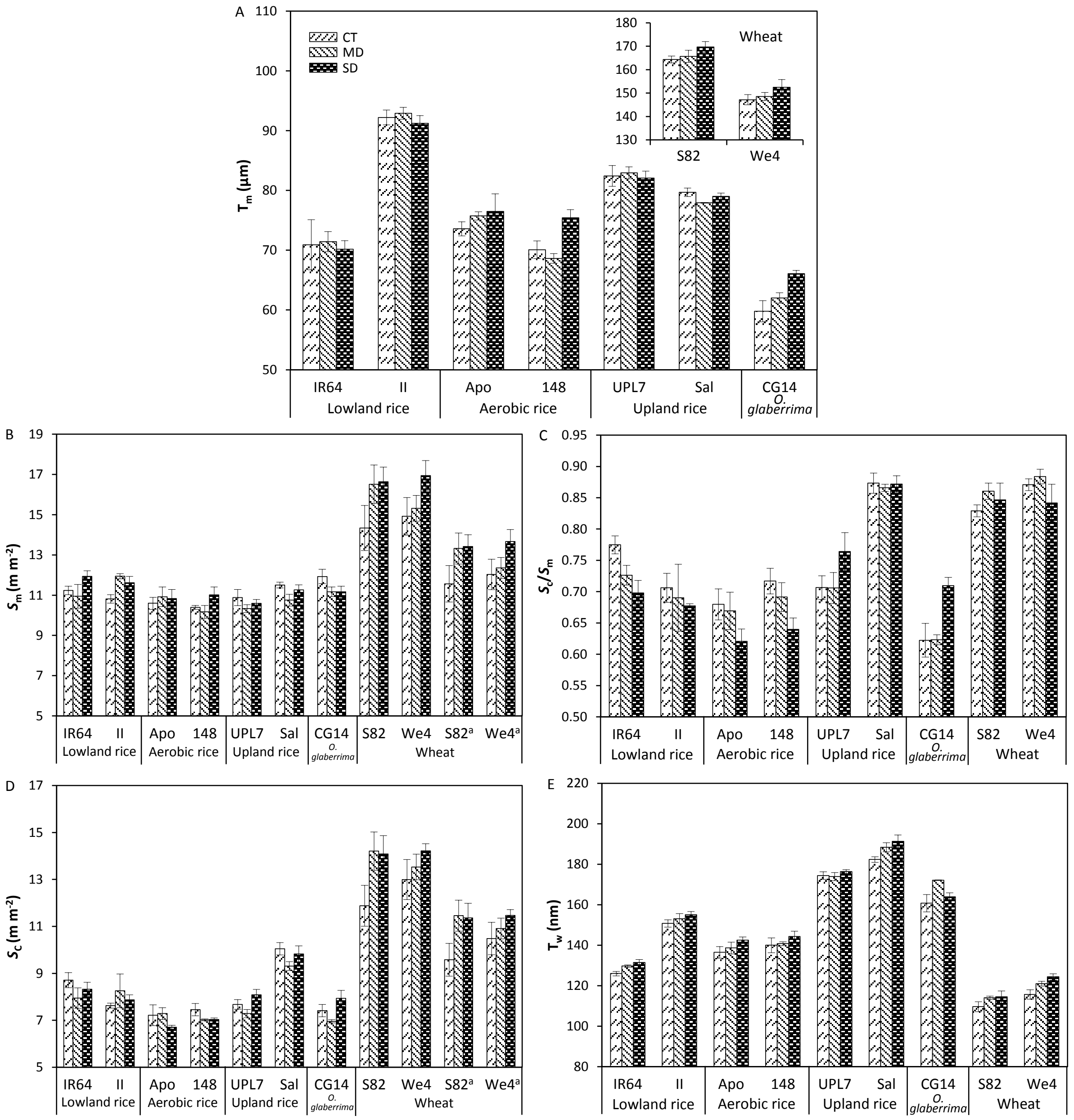

Fig. S5. Mesophyll cell properties of wheat and rice leaves obtained from light and electron microscope images under three treatments: control (CT); mild drought (MD); and more severe drought (SD). (A) mesophyll thickness (T_m); (B) the surface area of mesophyll cells exposed to the intercellular airspaces per leaf area (S_m); (C) ratio of the exposed surface area of chloroplast to the exposed surface area of mesophyll cell walls (S_c/S_m); (D) the surface area of chloroplasts exposed to intercellular airspace per leaf area (S_c); and (E) thickness of the mesophyll cell wall (T_w). For wheat cultivars marked by superscript [a] in B and D, we used the alternative value 1.25 as the curvature correction factor (F) for calculating S_m and S_c.

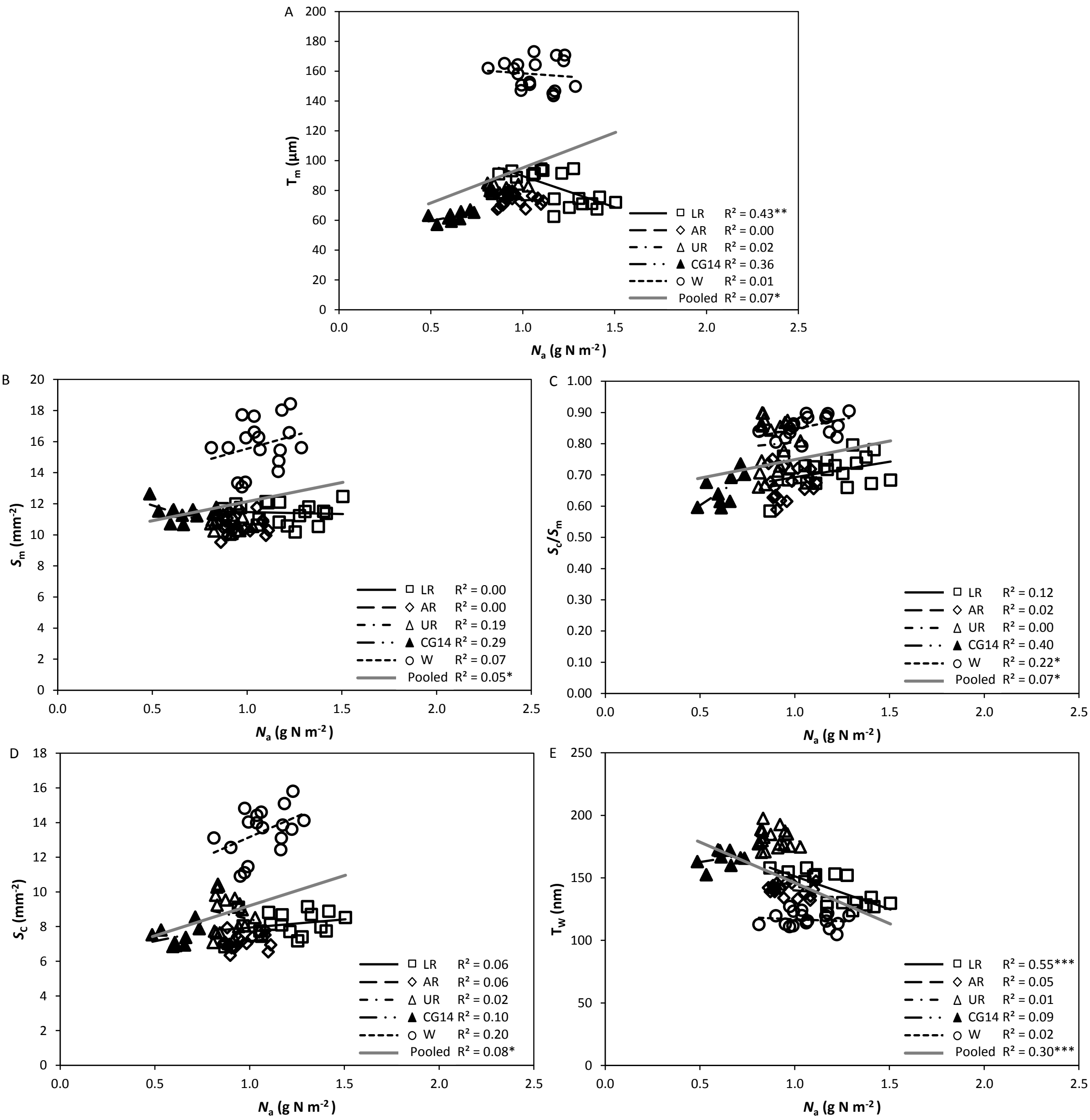

Fig. S6. Relationships between (A) mesophyll thickness (T_m) and N_a; (B) the surface area of mesophyll cells exposed to the intercellular airspaces per leaf area (S_m) and N_a; (C) ratio of the exposed surface area of chloroplast to the exposed surface area of mesophyll cell walls (S_c/S_m) and N_a; (D) the surface area of chloroplasts exposed to intercellular airspace per leaf area (S_c) and N_a; and (E) thickness of the mesophyll cell wall (T_w) and N_a. LR: lowland rice; AR: aerobic rice; UR: upland rice; CG14: *O. glaberrima*; W: wheat. Linear regressions were fitted for each genotype group. The significance of each correlation is shown as: *, P< 0.05; **, P< 0.01; ***, P<0.001.

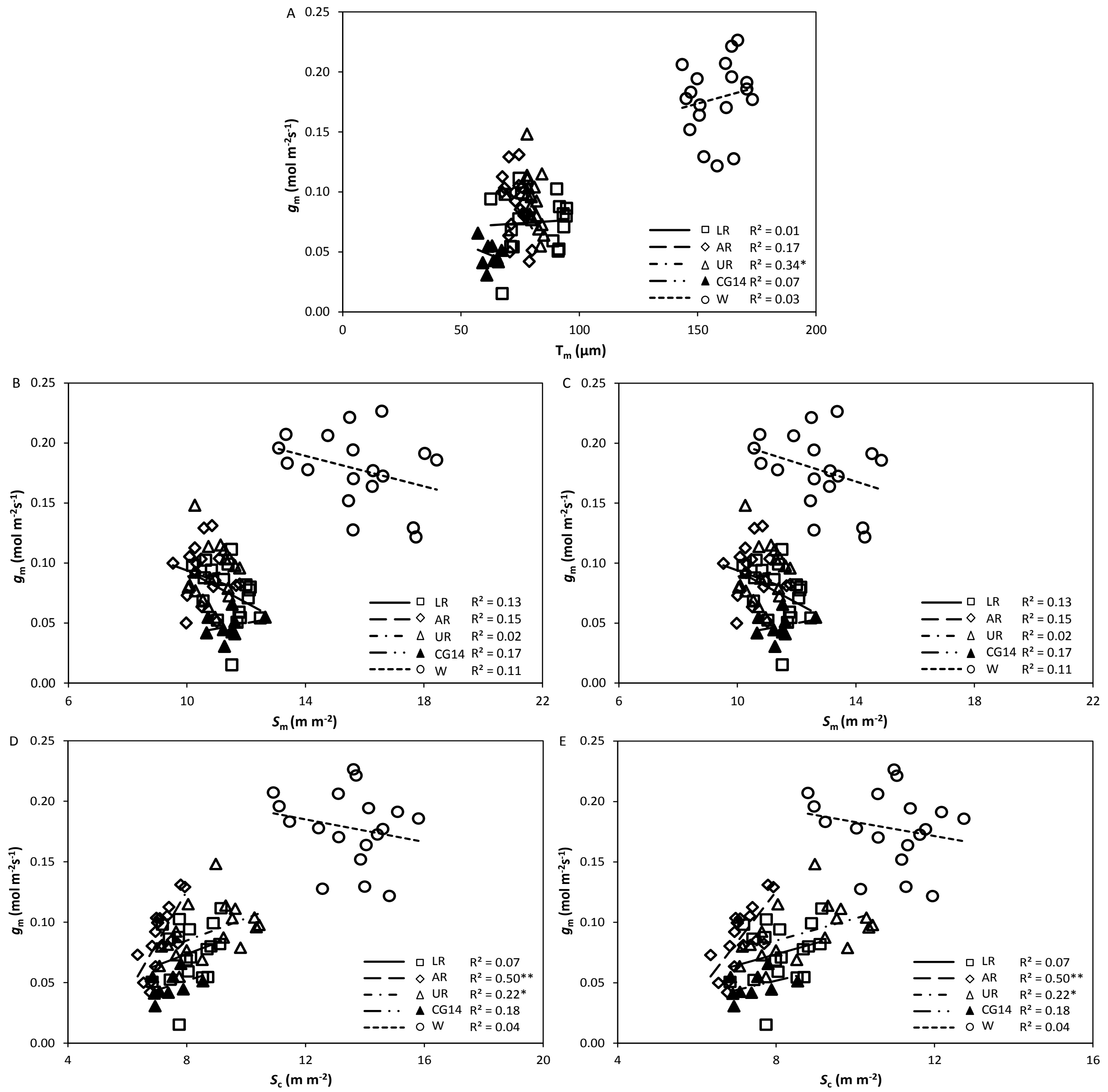

Fig. S7. The relationship between mesophyll conductance (g_m) (NRH-A method) and (A) mesophyll thickness (T_m); (B,C) the surface area of mesophyll cells exposed to the intercellular airspaces per leaf area (S_m); and (D,E) the surface area of chloroplasts exposed to intercellular airspace per leaf area (S_c). In B and D we used the value 1.55 as curvature correction factor (*F*) for calculating S_m and S_c in both rice and wheat cultivars; in C and E we used the value 1.55 as *F* for calculating S_m and S_c in rice and 1.25 for wheat cultivars. LR: lowland rice; AR: aerobic rice; UR: upland rice; CG14: *O. glaberrima*; W: wheat. Linear regressions were fitted for each genotype group. The significance of each correlation is shown as: *, P< 0.05; **, P< 0.01; ***, P<0.001.
